# Origin and diversification of the plasminogen activation system among chordates

**DOI:** 10.1186/s12862-019-1353-z

**Published:** 2019-01-17

**Authors:** Andrés Chana-Muñoz, Agnieszka Jendroszek, Malene Sønnichsen, Tobias Wang, Michael Ploug, Jan K. Jensen, Peter A. Andreasen, Christian Bendixen, Frank Panitz

**Affiliations:** 1 Department of Molecular Biology and Genetics, Aarhus University, 8830 Tjele, Denmark; 2 Department of Molecular Biology and Genetics, Aarhus University, 8000 Aarhus, Denmark; 3 Institute for Bioscience Zoophysiology, Aarhus University, 8000 Aarhus, Denmark; 4 Finsen Laboratory, Rigshospitalet, DK-2200 Copenhagen N, Denmark and Biotech Research and Innovation Centre (BRIC), University of Copenhagen, DK-2200 Copenhagen, Denmark; 5 Present address: Interdisciplinary Nanoscience Center – INANO-MBG, Aarhus University, 8000 Aarhus, Denmark

**Keywords:** Plasminogen, Plasminogen activation system, Evolution, Phylogenetic analysis, Chordates, Transcriptome analysis

## Abstract

**Background:**

The plasminogen (PLG) activation system is composed by a series of serine proteases, inhibitors and several binding proteins, which together control the temporal and spatial generation of the active serine protease plasmin. As this proteolytic system plays a central role in human physiology and pathophysiology it has been extensively studied in mammals. The serine proteases of this system are believed to originate from an ancestral gene by gene duplications followed by domain gains and deletions. However, the identification of ancestral forms in primitive chordates supporting these theories remains elusive. In addition, evolutionary studies of the non-proteolytic members of this system are scarce.

**Results:**

Our phylogenetic analyses place lamprey PLG at the root of the vertebrate PLG-group, while lamprey PLG-related growth factors represent the ancestral forms of the jawed-vertebrate orthologues. Furthermore, we find that the earliest putative orthologue of the PLG activator group is the hyaluronan binding protein 2 (HABP2) gene found in lampreys. The prime plasminogen activators (tissue- and urokinase-type plasminogen activator, tPA and uPA) first occur in cartilaginous fish and phylogenetic analyses confirm that all orthologues identified compose monophyletic groups to their mammalian counterparts. Cartilaginous fishes exhibit the most ancient vitronectin of all vertebrates, while plasminogen activator inhibitor 1 (PAI-1) appears for the first time in cartilaginous fishes and is conserved in the rest of jawed vertebrate clades. PAI-2 appears for the first time in the common ancestor of reptiles and mammals, and represents the latest appearing plasminogen activator inhibitor. Finally, we noted that the urokinase-type plasminogen activator receptor (uPAR)—and three-LU domain containing genes in general—occurred later in evolution and was first detectable after coelacanths.

**Conclusions:**

This study identifies several primitive orthologues of the mammalian plasminogen activation system. These ancestral forms provide clues to the origin and diversification of this enzyme system. Further, the discovery of several members—*hitherto* unknown in mammals—provide new perspectives on the evolution of this important enzyme system.

**Electronic supplementary material:**

The online version of this article (10.1186/s12862-019-1353-z) contains supplementary material, which is available to authorized users.

## Background

Plasminogen activation (PA) leads to the generation of the broad spectrum serine protease plasmin, which plays a primary role in fibrin surveillance and in securing vascular patency in species with a circulatory system. Other extracellular matrix (ECM) components are also targets for plasmin-mediated proteolysis, either directly or indirectly through activation of e.g. metalloproteinases [1]. In mammals, two main physiological plasminogen activators are known: tissue-type plasminogen activator (tPA; with the gene denoted *PLAT*) and urokinase plasminogen activator (uPA; with the gene denoted *PLAU*), both belonging to the serine-protease family. These activators have evolved different preferred compartments for their plasminogen activation potential: tPA is believed to act primarily on polymerized fibrin matrices resolving fibrin deposits in the vasculature, while uPA focalizes plasminogen activation onto cell surfaces, such as activated macrophages and neutrophils, thus providing a cellular component for fibrin surveillance in extravascular compartments and for ECM remodeling [1–4]. The confinement of uPA-mediated plasminogen activation to the pericellular compartment is driven by expression of the uPA receptor (uPAR; with the gene denoted *PLAUR*), which is a glycolipid-anchored membrane receptor [5] composed by three Ly6/uPAR or LU domains [6, 7]—all participating in the assembly of a high-affinity binding site for uPA [8–10]. uPAR interacts weakly with the somatomedin-B (SMB) domain of the provisional matrix protein vitronectin (VN) [11, 12], providing an additional layer to the regulation of cell attachment and migration. This process is under allosteric control by the high-affinity binding of uPA [13, 15, 16]. The main physiological inhibitor of tPA and uPA is a circulating serpin (serine protease inhibitor) termed plasminogen activator inhibitor-1 (PAI-1) [17]. The PAI-1 inhibitory mechanism is common to other serpins in which the exposed reactive center loop (RCL) of the serpin is recognized as a substrate for the target proteases. After proteolytic cleavage of the RCL the serpin undergoes a large conformational change, the so-called stressed-to-relaxed transition, which inactivates the protease and leaves the RCL no longer accessible [18]. Contrary to other serpins, PAI-1 can spontaneously undergo the stressed-to-relaxed transition, adopting a latent inactive state (latency transition). Interestingly, the binding of PAI-1 to its cofactor VN increases the half-life of the active form of PAI-1 [18–20]. Due to the importance of the plasminogen activation system (PAS) in human health and disease, it has been extensively studied in mammals and been a popular target for drug development [14, 21, 22]. Although only a limited number of studies of this system exist in other vertebrates [23–28], they have suggested that the mechanisms for plasminogen activation and inhibition are more complex than the one emerging from merely reconciling observations made in mammals.

**Fig. 1:**
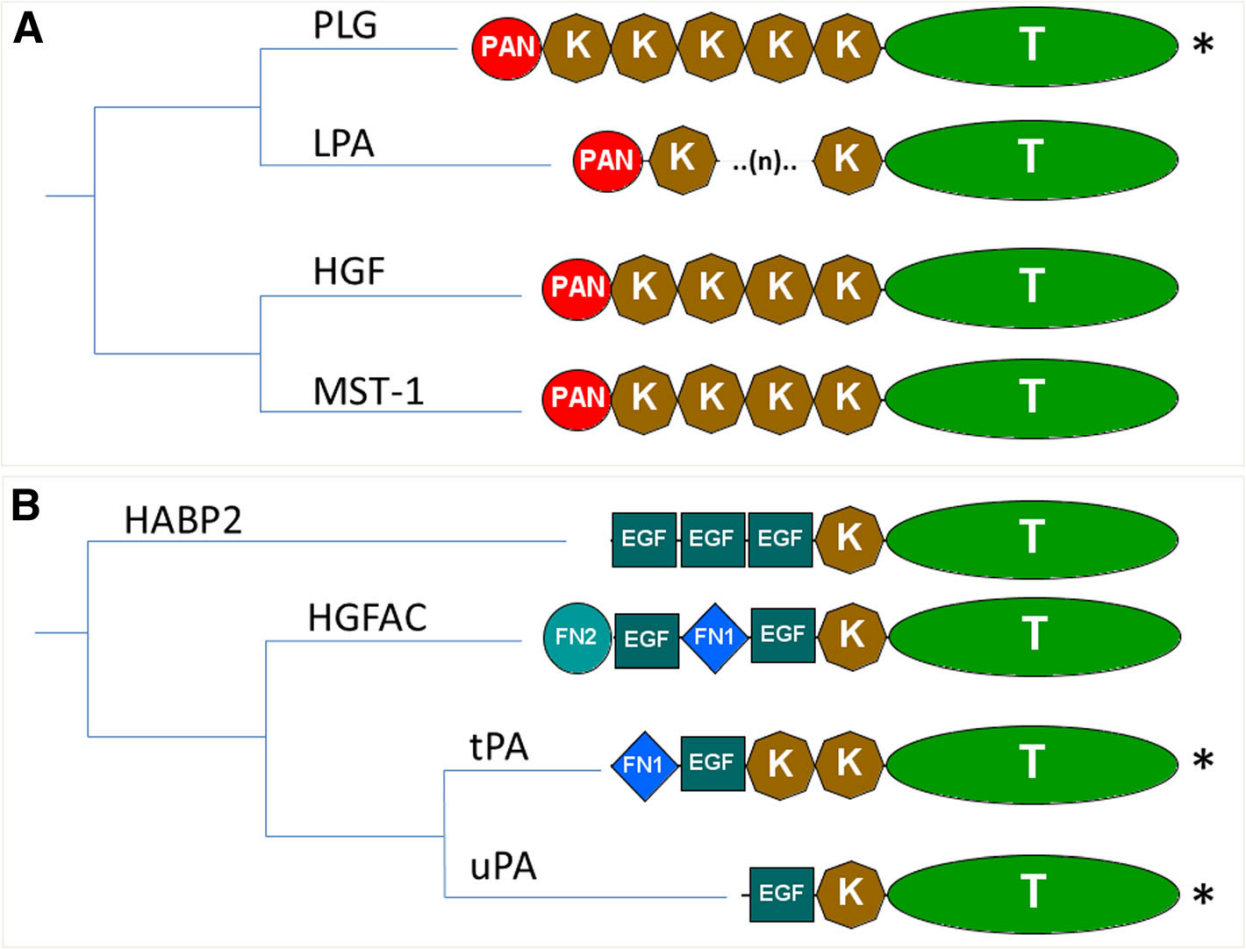
Phylogenetic relationship and protein domain composition of the human serine protease members of the plasminogen activation system and their close paralogues. **a** PLG-group and **b** PLG activators group members. PLG (plasminogen), HGF (hepatocyte growth factor), MST-1 (macrophage stimulating 1), HABP2 (hyaluronan binding protein 2), LPA (lipoprotein a), HGFAC (hepatocyte growth factor activator), tPA (tissue-type plasminogen activator) and uPA (urokinase-type plasminogen activator). (*) Members of PAS in mammals. Domain composition: FN1 (fibronectin type 1), FN2 (fibronectin type 2), K (kringle), EGF (epidermal growth factor), PAN (PAN/APPLE), T (trypsin). Phylogenetic tree derived from the human phylome from PhylomeDB [36]

Plasminogen and the serine proteases of the PAS are believed to have emerged from a common ancestral gene which further diverged into the present members of the PLG-group and PA-group [29–36] (Fig. 1). The existence of a number of protein domains shared by those extant members prompted the development of several theories about the evolutionary origin of these serine proteases [31, 33–35, 37], which are summarized in Additional file 1: Figure S1A and Figure S1B. Although there is disagreement concerning the domain composition of the common ancestor gene, it is generally accepted that the current members arose as a consequence of exon-shuffling events combined with gene duplications [29, 31, 33, 34]. The main plasminogen activator inhibitor PAI-1, also termed SERPINE1, is composed by a single serpin domain and belongs to the V3 serpin group with its close paralogues SERPINE2, SERPINE3 and SERPINI1 [38–41]. However, the shutter region of PAI-1 exhibits an unique residue composition, which allows a sequence based discrimination of PAI-1 from other related serpins [19, 26, 42]. Another feature of PAI-1 is its binding to the somatomedin-B domain of VN [43], which is comprised of SMB, an integrin binding motif, and four Hemopexin-like (HX) repeats. Although the presence of VN in several mammalian and avian species was characterized in an earlier study [44], large scale comparative studies on VN are lacking.

In addition to PAI-1, VN also interacts with uPAR, which belongs to the Ly6/uPAR family [6]. This protein domain family is characterized by the presence of at least one LU domain with 10 conserved cysteine residues. These conserved residues are involved in the formation of five disulphide bonds creating the characteristic three-fingered fold found in the primordial snake venom α-neurotoxins [6]. Mammalian uPAR possess three of those LU domains, which are all required for its biological function [10, 16, 45]. While these disulfide bonds are absolutely essential for the correct protein folding of the single LU domain proteins [45–47], the first LU domain in uPAR unexpectedly lacks one of these consensus disulfide bonds and only presents eight cysteines [10, 48, 49]. This feature is shared among the multi-LU-domain paralogues C4.4A, Haldisin, TEX101, CD177, and PINLYP [48, 50–53]. However, information on LU-containing genes in non-mammalian vertebrates is very limited.

Previous comparative studies of the plasminogen activation system focused on the serine proteases, which are believed to descend from a common ancestor gene by gains and losses of protein domains. These studies were performed in a limited number of chordate species and the detection of transitional forms providing direct evidence of their common origin remains elusive. Despite the fact that due to the ongoing development of next-generation sequencing (NGS) technologies the number of genomes available in various sequence databases is increasing, sequence data from several vertebrate groups are still rather sparse.

**Fig. 2:**
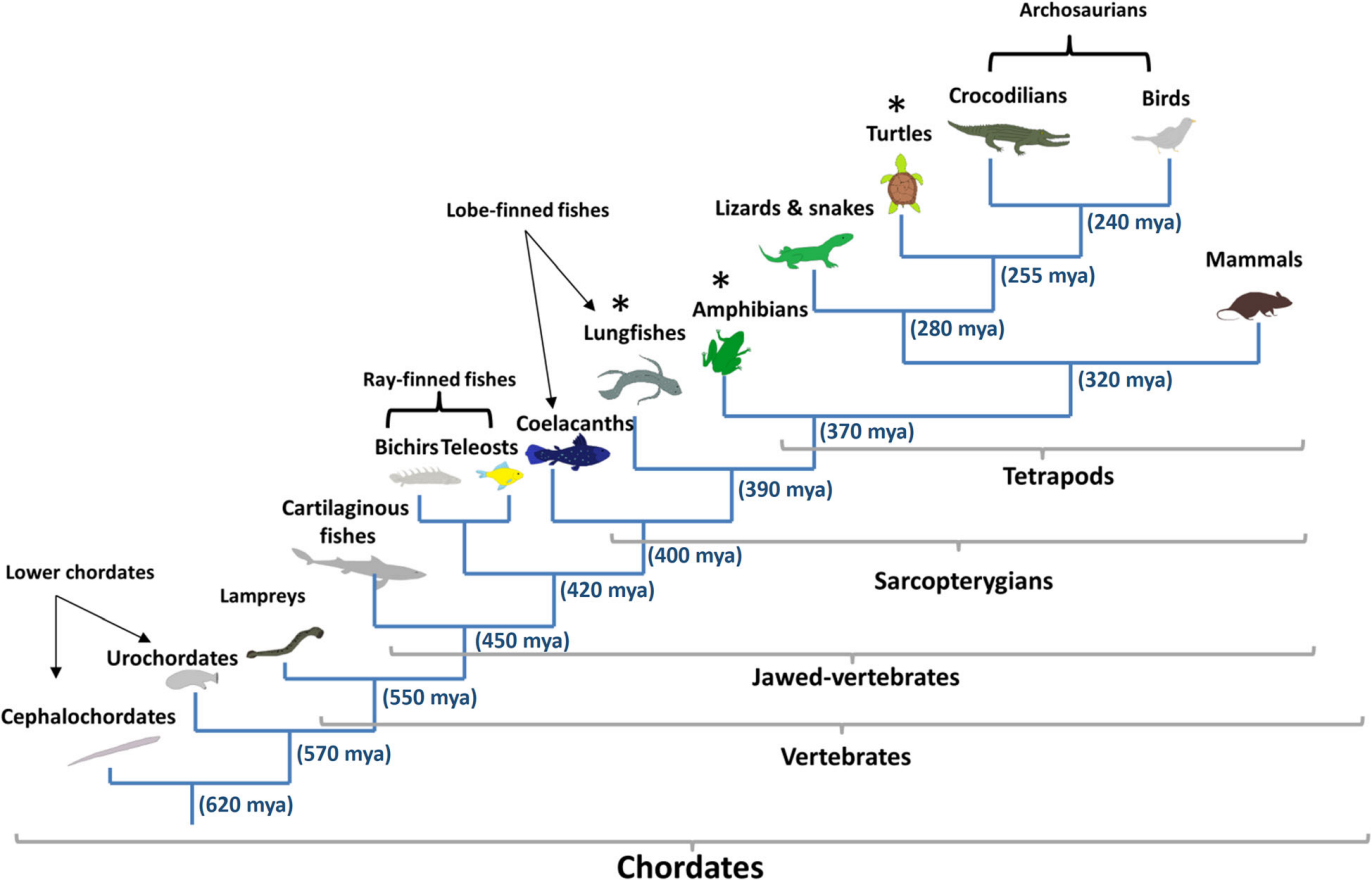
Phylogeny of the chordate main groups. Vertebrate groups as described in [54] from which new data was generated (*). Approximate divergence dates are expressed in million years ago (mya) and were collected from [54–59]

To overcome these limitations, we performed a comprehensive survey of genes involved in PAS by combining transcriptome and genome data from 13 different chordate groups [54–61] corresponding to 110 different species (Fig. 2). In addition, we performed extensive transcriptome sequencing in three vertebrate species belonging to groups with limited sequence information (lungfishes, amphibians and turtles). Our work provides the first large-scale comparative study of PAS members and their close paralogues among chordates. We confirm the presence of plasminogen among all vertebrate classes and tracked the origin of PAS to jawed-vertebrates. Moreover, we report the identification of ancestral forms of PAS members in lower vertebrates with no human equivalent, providing new insights into the development of this complex enzyme system.

## Results

### RNA-seq, de novo assembly and generation of protein sequence database from public repositories

Around 617 and 327 million 150 bp paired-end reads were generated from brain, kidney, liver and gonads from African lungfish (*Protopterus sp)* and pond slider turtle (*Trachemys scripta),* respectively, while 98 million 150 bp paired-end reads were sequenced from kidney and liver from a cane toad (*Rhinella marina)*. After the initial data processing, de novo transcriptome assemblies were performed using Trinity [62, 63]. Following filtering, the transcriptomes were annotated with the Trinotate pipeline (https://trinotate.github.io; see Methods). An overview of the annotation results is contained in Table 1.

**Table 1:** Assembly metrics and transcriptome annotation of the RNA-seq data generated

Raw assembly metrics			
Feature	*Protopterus sp*	*Rhinella marina*	*Trachemys scripta*
Trinity genes	1,241,097	289,963	972,365
Trinity transcripts	1,423,377	256,160	850,085
N50	362 bp	513 bp	559 bp
Mapped reads	^a^65% proper	^a^70% proper	^a^65% proper
	^b^12% single	^b^10% single	^b^15% single
^c^Completeness	83%	54%	81%
^d^After filtering			
Feature	*Protopterus sp*	*Rhinella marina*	*Trachemys scripta*
Trinity genes	395,129	110,639	464,338
Trinity transcripts	458,605	125,853	540,638
N50	794 bp	859 bp	745 bp
^c^Completeness	82%	54%	80%
Predicted peptides	143,312	50,831	133,635
SwissProt	69,155	36,468	77,940
Uniref90	82,471	38,551	84,921
Pfam	55,446	27,460	55,196
SignalP	6449	2887	6686
TMHMM	16,366	6441	14,712
EggNOG	35,778	16,017	38,348
GO	97,438	34,074	70,948

^a^Both read pairs mapped to the same transcript

^b^One of the reads from the pair mapped to a different transcript

^c^Completeness by BUSCO analysis [98] against a set of core vertebrate conserved genes

^d^Filtering performed by transcript length and expression level (see Methods)

Additionally, 296 million reads from a catfish species (*Pangasius hypophthalmus*) were sequenced and assembled into 393,517 contigs providing 81,349 predicted proteins for that species (data not shown). The total pool of species examined was increased by using publicly available RNA-seq reads (see Methods and Additional file 2), assembled data from public RNA-seq databases, two transcriptome assemblies previously performed by our group [64] and data derived from public available transcriptome and genome assemblies (Additional file 2).

### Emergence of the PLG-group

In this study we focused on PLG and orthologues of the PLG-related growth factors (Fig. 1), which have lost their catalytic activity [20]. Primate genes encoding lipoprotein (a) (LPA) with a highly variable number of kringle repeats [65–67] were, however, excluded from the analysis.

**Fig. 3:**
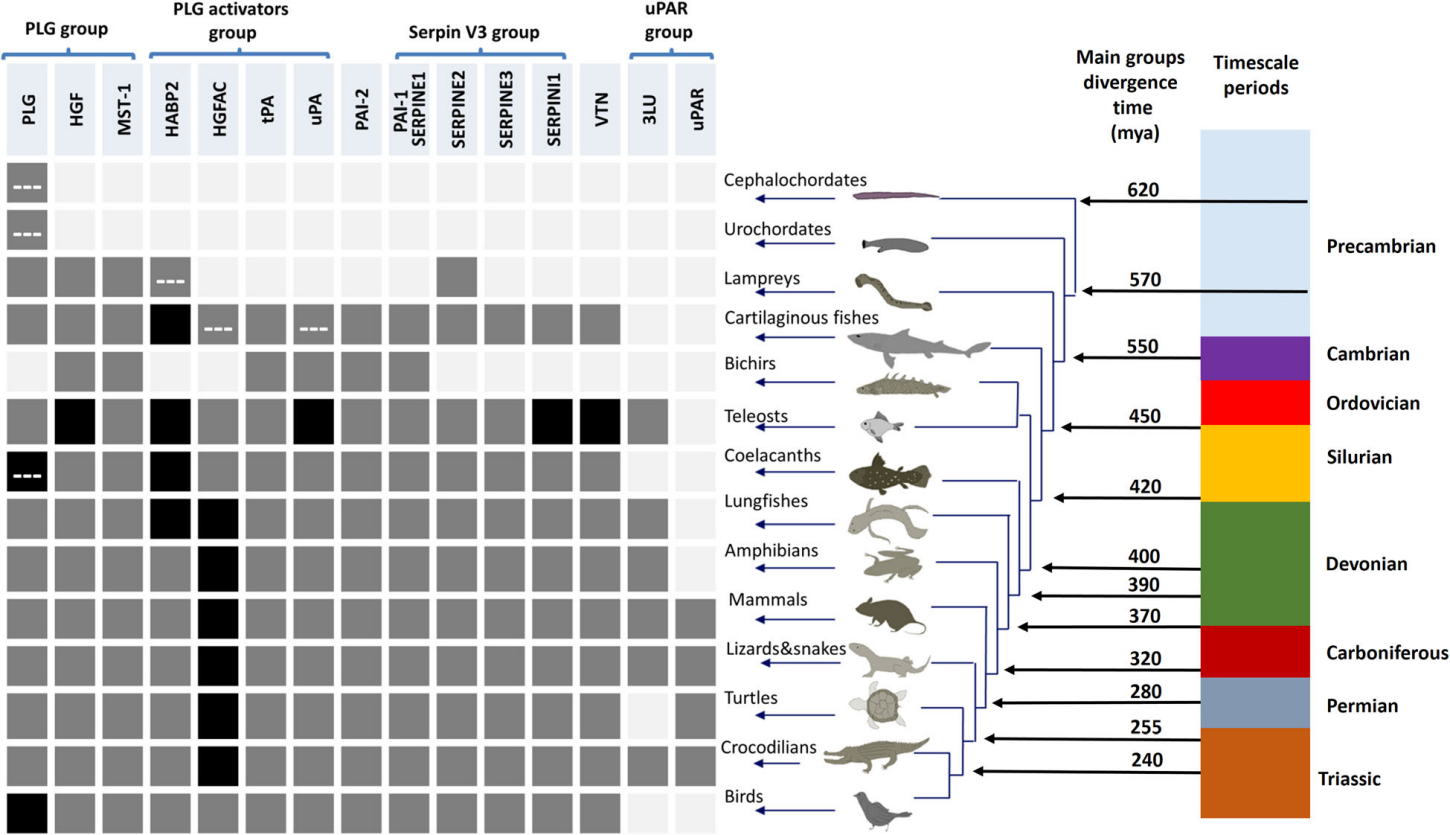
Presence of plasminogen activation system members and related paralogues among the chordate species investigated. Squares indicate absence (light grey), presence (dark grey) and duplication (black) of the different orthologues. Dashed-lines (---) indicate non-canonical protein domain composition. Approximate divergence time in million years ago (mya) was collected from [54–59]. PLG (plasminogen), HGF (hepatocyte growth factor), MST-1 (macrophage stimulating 1), HABP2 (hyaluronan binding protein 2), HGFAC (hepatocyte growth factor activator), tPA (tissue-type plasminogen activator) and uPA (urokinase-type plasminogen activator), VTN (vitronectin), uPAR (urokinase-typle plasminogen activator receptor), LU (Ly6/LU domain)

**Fig. 4:**
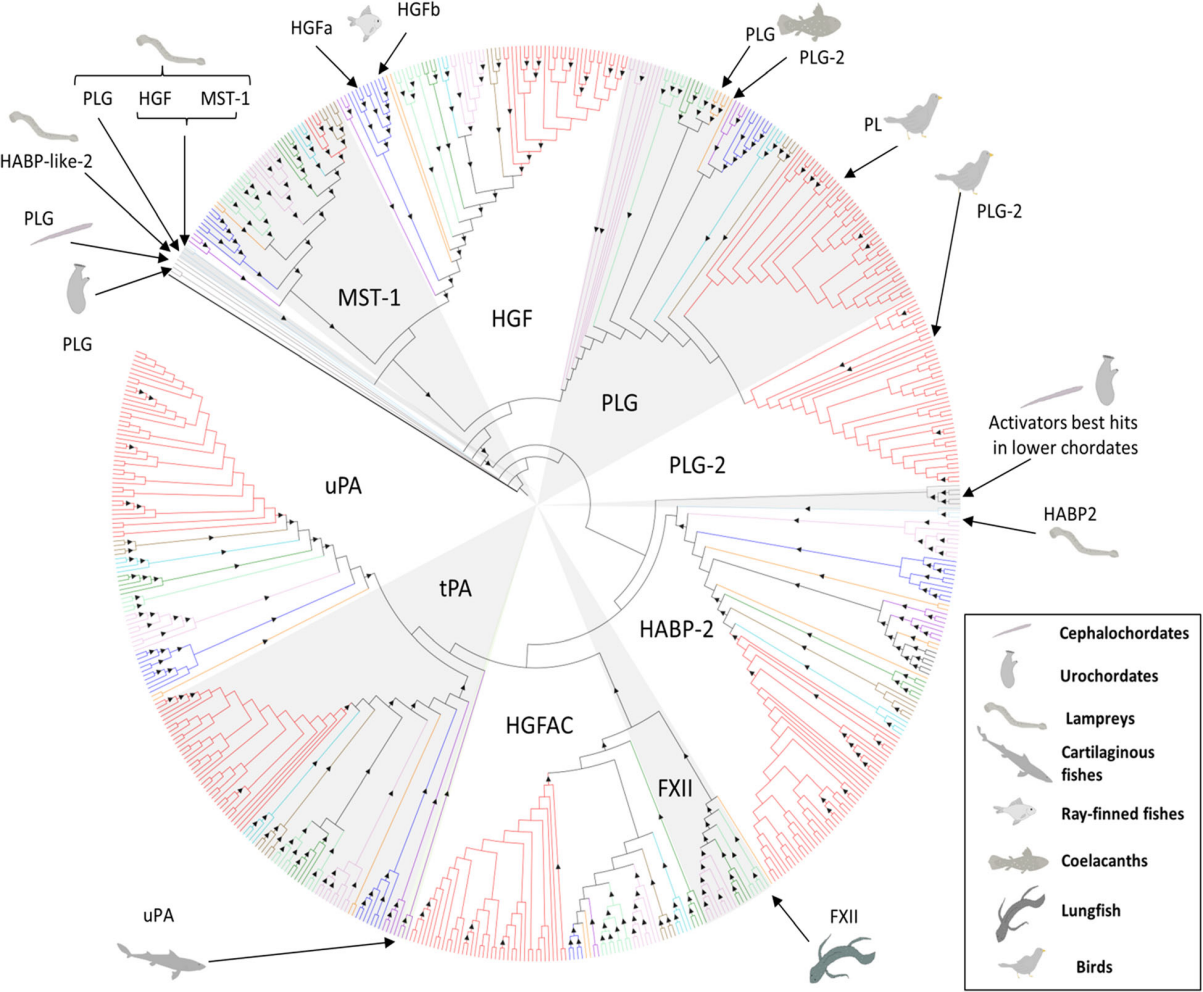
Maximum-likelihood phylogenetic tree of the PLG and PLG activator groups of serine proteases. *Caenorhabditis elegans* SVH-1 (BAL45941.1) was chosen as outgroup to root the tree. Triangles within branches represent bootstrap support higher than 50% after 1000 replicates. Colored branches indicate lower chordates (grey), cartilaginous fishes (violet), ray-finned fishes (blue), coelacanths and lungfish (orange), amphibians (light green), lizards and snakes (dark green), turtles (turquoise), crocodilians (brown) and birds (red). Position of the non-canonical or first appeared candidates is indicated within the tree by arrows. PLG-2 corresponds to the extra PLG gene identified, which has lost the catalytic activity. PLG (plasminogen), HGF (hepatocyte growth factor), MST-1 (macrophage stimulating 1), HABP2 (hyaluronan binding protein 2), HGFAC (hepatocyte growth factor activator), tPA (tissue-type plasminogen activator), uPA (urokinase-type plasminogen activator) and FXII (coagulation factor XII/Hageman factor). The unrooted tree with bootstrap values is provided in Additional file 3

After orthology assignment, our results show that in lower chordates the only PLG-like proteases found (Fig. 3) comprise no more than two to three kringle domains attached to the trypsin-like domain (Additional file 1: Figure S2)*.* On the other hand, lampreys (cyclostomes) present a PLG orthologue with a domain composition similar to that of their mammalian counterparts. The appearance of this protease is coupled with the emergence of the two plasminogen related growth factors HGF (hepatocyte growth factor) and MST-1 (macrophage stimulating 1), which already in lampreys are predicted to have lost their catalytic activity (Additional file 1: Figure S3). The presence of PLG, HGF and MST-1 is conserved in the rest of the vertebrate groups examined (Fig. 3). However, a special feature is found in two coelacanth species where the PLG orthologues have lost the PAN (PAN/APPLE) domain, two of the three catalytic sites in the trypsin domain and potentially the catalytic activity (Additional file 1: Figure S3). A detailed examination revealed several gene duplications in vertebrates subsequent to their emergence in lampreys. As seen in Fig. 3 and Fig. 4, teleosts present two HGF genes, while coelacanths and birds have two PLG copies, which in the case of coelacanths contain only one kringle domain and no catalytic activity (Additional file 1: Figure S3). Finally, phylogenetic analysis locate lamprey PLG at the root of the whole vertebrate PLG-group while both lamprey PLG-related growth factors are positioned at the root of the HGF-MST-1 group representing the ancestral forms of the jawed-vertebrate orthologues (Fig. 4 and Additional file 3).

### Emergence of the PLG activator group

According to previous phylogenetic studies of this gene group, HABP2 (hyaluronan binding protein 2) split from the main branch before the appearance of a common uPA and tPA ancestral gene (Fig. 1). HABP2, also termed factor seven-activating protein (FSAP), was first implicated in coagulation, but later its ability to activate uPA was reported as well [68]. Interestingly, HABP2 can be inhibited by PAI-1 [69] and SERPINE2 [70]. Prompted by these observations, we performed an exhaustive search including all the members of the PLG activators group to study the evolution of plasminogen activation.

Our analysis showed that none of the six species belonging to the lower chordates seem to have an obvious orthologue to the PLG activator group as their obtained Blast reciprocal best hits (BRBH) displayed several protein domains not known to occur in their mammalian counterparts (Table 2). Nonetheless, all of them are located at the root of the PLG activators group (Fig. 4), with 41% bootstrap support after 1000 replicates (Additional file 3) confirming their close phylogenetic relationship.

**Table 2:** Reciprocal best hits (BRBH) to human PLG activator group members in lower chordates

Animal group	Species	BRBH	Protein domain types
Cephalochordates	*Branchiostoma belcheri*	tPA	SRCR, Kringle, LDLRA, PAN, Trypsin^a^
		HABP2	SRCR, Kringle, LDLRA, PAN, Trypsin
	*Branchiostoma floridae*	HGFAC	SRCR, Kringle, LDLRA, Trypsin^a^
	*Assymetron lucayanum*	HABP2	SRCR, Kringle, LDLRA, PAN, Trypsin
Urochordates	*Ciona intestinalis*	HABP2	FN2, PAN, Kringle, Trypsin
	*Oikopleura dioica*	HABP2	Kringle, Trypsin

^a^indicates Trypsin domain predicted to be non-functional. SRCR (scavenger receptor cysteine-rich domain), LDLRA (Low-density lipoprotein (LDL) receptor class A), FN2 (fibronectin type 2 domain)

The earliest putative orthologue of the PLG activator group, resembling the mammalian equivalent tPA, is the HABP2 gene found in lampreys, presenting an extra fibronectin type 2 (FN2) domain in its N-terminal region compared to their mammalian counterpart (Additional file 1: Figure S2). From cartilaginous fish to birds the canonical forms of HABP2 are found through all vertebrate clades, which cluster together with the lamprey HABP2 in a monophyletic group (Fig. 4). Non-tetrapod jawed vertebrates display an additional gene resembling HABP2 (Fig. 3).

In the case of the hepatocyte growth factor activator (HGFAC), the most primitive orthologue is present in cartilaginous fish (elasmobranchs). Strikingly, HGFAC in elasmobranchs displays twice as many epidermal growth factor- (EGF), fibronectin-1 (FN1) and kringle domains as compared to the mammalian HGFAC (Additional file 1: Figure S2). Canonical HGFAC forms are found from teleosts throughout all the vertebrate clade, and the HGFAC duplication, which led to the appearance of coagulation factor XII (FXII; Hageman factor) in mammals, took place before the emergence of lungfishes (Fig. 3). The HGFAC and FXII precursor forms we detected form a monophyletic group, which further branches into HGFAC and FXII groups (Fig. 4).

**Fig. 5:**
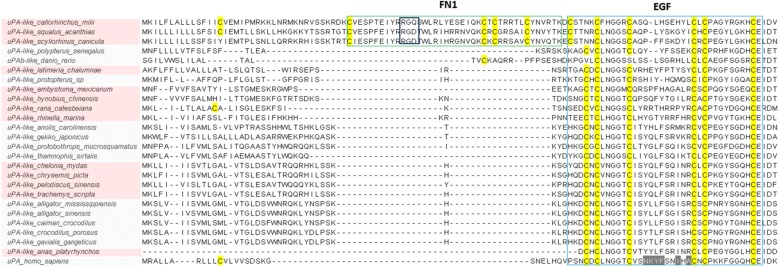
Multiple sequence alignment of the N-terminal region of selected vertebrate uPAs. uPA (urokinase-type plasminogen activator), FN1 (fibronectin type 1), EGF (epidermal growth factor)

With respect to tPA and uPA, they both first occur in cartilaginous fish (Fig. 3). In jawed-vertebrates, tPA possesses a domain composition identical to the human equivalent, while the ancestral uPA in cartilaginous fish deviates from the canonical domain composition (Additional file 1: Figure S2). It possesses an extra FN1 domain in the N-terminal part, which in elasmobranchs interestingly displays an integrin-binding motif (Fig. 5). Teleosts present two uPA genes (a and b), as previously described [24]. However, the bichir *Polypterus senegalus* (a non-teleost ray-finned fish) exhibits only one uPA gene with a complete EGF domain in the N-terminal part thus resembling mammalian uPAs (Fig. 5). In the remaining jawed-vertebrates, uPA orthologues display the canonical domain composition. Finally, phylogenetic analyses confirm that all uPA and tPA orthologues identified compose monophyletic groups to their mammalian counterparts, with the exception of the ancestral uPAs found in cartilaginous fishes located at the root of both activators (Fig. 4).

### Emergence of vitronectin, PAI-1 and related paralogues

Our results showed that cartilaginous fishes exhibit the most ancient VN of all vertebrates displaying a SMB domain, an integrin binding region and the four HX-domain repeats—thus resembling the composition of the human orthologue. The presence of VN is conserved in all the examined jawed vertebrate groups, with the appearance of an extra gene copy (a and b) in teleosts (Additional file 1: Figure S4).

Although PAI-1 is the predominant PA inhibitor in humans, its close paralogues SERPINE2 (glia-derived nexin) [71] and SERPINI1 (neuroserpin) are indeed capable of inhibiting PA activity as well [72–74]. Based on this reasoning, the orthologues of SERPINE2 and SERPINI1 were included in the study. Furthermore, SERPINE3 and SERPINB2 genes were also investigated, as SERPINE3 is the only close paralogue of PAI-1 without a known function, and plasminogen activator inhibitor-2, placental type (PAI-2)—albeit distantly related to PAI-1—is believed to be a specific PA inhibitor in human placental tissue [75].

**Fig. 6:**
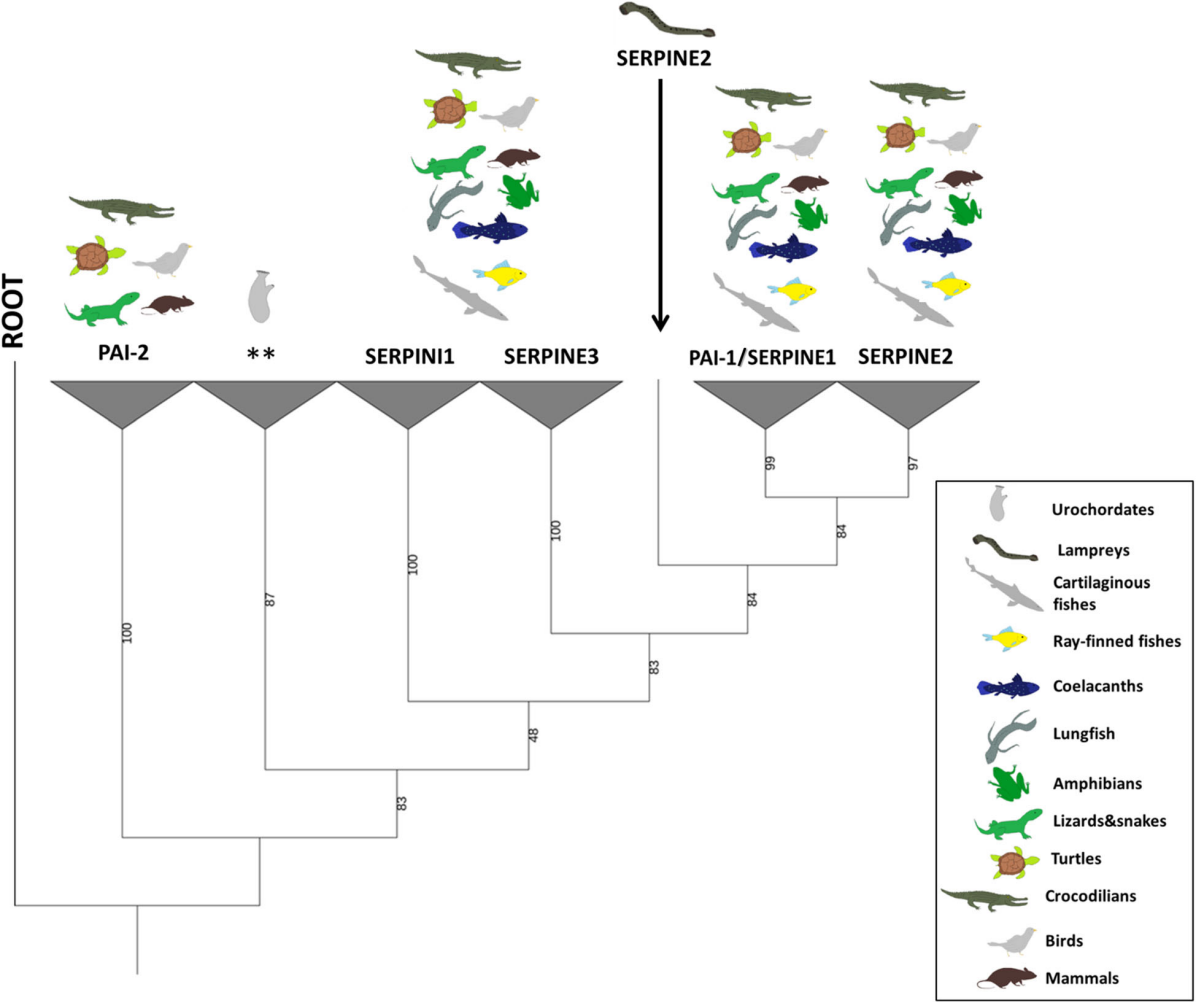
Phylogenetic relationship serpin V3 members and PAI-2. Maximum-likelihood tree rooted at SPN-1 *Nematostella vectensis* (XP_001627732.1). Percentage of bootstrap support after 1000 replicates shown in the branches. Asterisks (**) indicate the location of reciprocal best hits to human PAI-1 and SERPINI1 in urochordates. PAI-1/SERPINE1 (plasminogen activator inhibitor-1/serpin E1), SERPINE3 (serpin E3), SERPINI1 (serpin I1), SERPINE3 (serpin E3), PAI-2 (plasminogen activator inhibitor-2, placental type). The unrooted tree is provided in Additional file 4

Amongst the lower chordates, only urochordates present BRBH to any of the five serpins investigated, in particular to PAI-1 and SERPINI1. Notwithstanding this putative relatedness, a more refined analysis revealed that although they cluster together in an independent group at the root of the serpin V3 members (Fig. 6 and Additional file 4) they do not present an obvious similarity with their vertebrate equivalents. Regarding vertebrates, SERPINE2 is the only member of the V3 serpin group found in cyclostomes (lamprey). PAI-1 appears for the first time in cartilaginous fishes and is conserved in the rest of jawed vertebrate clades.

In addition, all the jawed vertebrate groups exhibit SERPINE3 and SERPINI1 orthologues, contrary to PAI-2, which appeared for the first time in the common ancestor of reptiles and mammals and represents the latest appearing PA inhibitor (Fig. 3). All identified candidates form monophyletic groups with their mammalian equivalents, except lamprey SERPINE2, which is located at the root of the SERPINE1-SERPINE2 group in jawed-vertebrates (Fig. 6).

### Appearance of uPAR and three-LU domain containing genes

In mammals, uPAR is the only known gene comprising three consecutive LU domains. Additional multi-LU genes known in this animal group are *LYPD3*/C4.4A [10], *LYPD5*/Haldisin [50], TEX101, PINLYP—containing two LU domains, and CD177—containing four LU domains [48]. Those mammalian multi-LU genes are located in a small gene cluster [7, 50, 52] in close proximity to other non-LU genes such as ETHE1, XRCC1 and PHLDB3. As the 3 LU-domain architechture of uPAR is a prerequisite for the assembly of a high affinity uPA binding cavity [8–10, 45], we included genes containing three LU domains, as well as BRBH of human uPAR, in the analysis of the different chordates species.

**Fig. 7:**
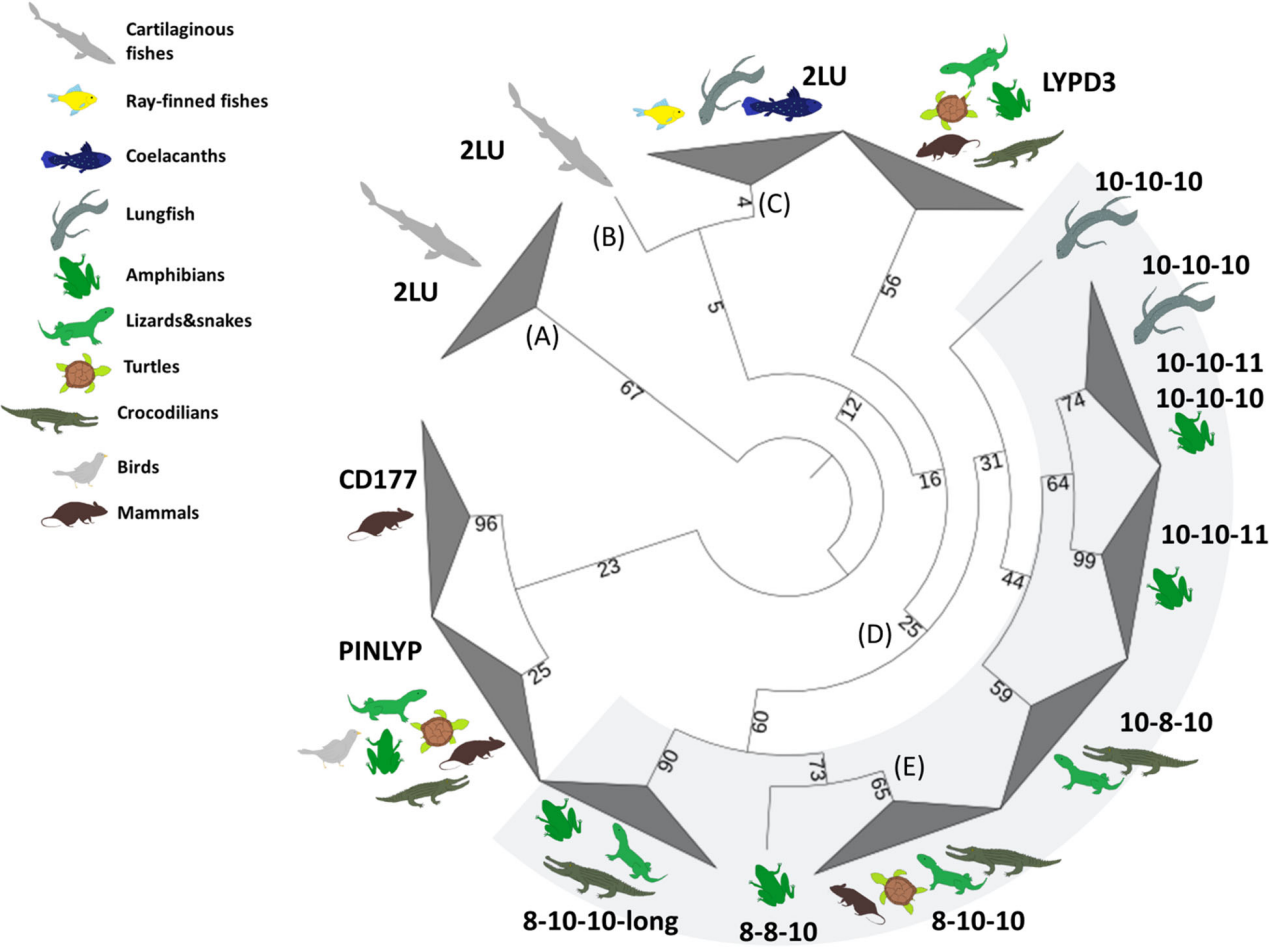
Phylogenetic tree of the different proteins containing three LU domains identified in vertebrates and close paralogues. Percentage of bootstrap support after 1000 replicates is shown in each branch. The tree was rooted at the elasmobranch 2 LU branch for convenience in display. **a** uPAR BRBH in elasmobranchs, **b** Elephant shark (cartilaginous fish) uPAR BRBH, **c** BRBH to ray-finned fishes uPAR and PINLYP, coelacanth uPAR and lungfish PINLYP. **d** Branch containing all the sarcopterygian three-LU genes. Numbers displaying the cysteine pattern of their first, second and third LU domains. **e** Branch containing human uPAR. uPAR (urokinase-type plasminogen activator receptor), LU (Ly6/uPAR domain), PINLYP (phospholipase A2 inhibitor and LY6/PLAUR domain containing protein), CD177 (cluster of differentiation 177)

As a general finding, we were unable to identify any three-LU domain-containing genes from cartilaginous fishes to coelacanths. The closest resemblance to the domain composition of uPAR comprised two-LU domain proteins, which may in fact be homologs of the corresponding 2-LU domain proteins found in the human genome. One exception is the genome of the teleost *Lates calcarifer*, which actually contains one three-LU domain gene as the best hit to human uPAR. Lungfish and most of the tetrapods species inspected possess more than one gene encoding a three-LU domain protein, with the exceptions of mammals, which possess only a single uPAR gene, and birds where a bona fide three-LU domain gene is absent (Fig. 3). Phylogenetic analysis showed all the identified candidates genes encoding a three-LU domain protein cluster close to *LYPD3* (which in mammals correspond to the closest paralogue to uPAR), but in a separate monophyletic group. The group containing a three-LU domain signature further branches into separate groups defined by variations in the cysteines patterns of their LU domains or the presence of a long stretch at the end of the third LU domain (Fig. 7, Table 3, Additional file 5 and Additional file 6). We also provide evidence that uPAR-like genes—identified in tetrapods and lungfish—all are located in close proximity to at least one of the genes present in the mammalian uPAR conserved gene cluster in those species where a genome assembly is available (Table 3). This provides strong evidence and support of their common evolutionary origin.

**Table 3:** Cysteine patterns of the different three-LU domain predicted proteins found in non-mammalian vertebrates

Animal group	Species	^a^uPAR BRBH	^b^Other three-LU
Ray-finned fishes	*Lates calcarifer*	10–10-10^e^	No
Lungfish	^c^ *Protopterus sp* ^f^	10–10-10	10–10-10
	*Nanorana parkerii*	10–10-11^d^	No
	*Xenopus tropicalis*	10–10-11^d^	No
Amphibians	*Xenopus laevis* ^e^	8–10-10-long^d^	10–10-10^d^ and 10–10-11^d^
	^c^ *Ambystoma mexicanum* ^f^	8–8-10	8–10-10-long, 10–10-1 and 10–10-11
	*Tylototriton wenxianensiss* ^f^	10–10	8–10-10-long, 10–10-11 and 10–10-11
	^c^ *Anolis carolinensis*	8–10-10^d^	8–10-10-long^d^
Lizards & Snakes	^c^ *Gekko japonicus*	8–10-10^d^	No
	^c^ *Protobothrops mucrosquamatus*	8–10-10^d^	No
	^c^ *Python bivittatus*	8–10-10	8–10-10-long and 10–8-10
Turtles	^c^ *Trachemys scripta* ^f^	8–10-10	No
Crocodilians	^c^ *Alligator mississippiensis*	8–10-10^g^	8–10-10-long and 10–8-10

^a^Best hit to human uPAR

^b^Other predicted peptides with three LU (Ly6/uPAR) domains

^c^Species with intact EGF in uPA

^d^Gene located in the same scaffold as at least one of ETHE1, XRCC1, PHLDB, LYPD3 or LYPD5

^e^Gene not located in the same scaffold as ETHE1, XRCC1, PHLDB, LYPD3 or LYPD5

^f^Genome assembly not available for that species

^g^Predicted protein obtained after merged two overlapping contigs from two different databases (see Additional file 2)

## Discussion

### PLG group orthologues (PLG and PLG-related growth factors)

A recent study reported a gene from the nematode *Caenorhabditis elegans* presenting both protease and growth factor-like activities [76] and being homologous to members of the PLG-group. This gene displays major differences in its domain composition compared to vertebrate PLG and is involved in axon regeneration. Notwithstanding these notable differences, it is believed to be a true PLG homologue arguing that the common ancestor of the PLG group may already present both functions [76]. Previous work also reported that two-kringle containing proteases with PLG-like activity constitute PLG orthologues in cephalochordates. This proposition aligns well with our present data. Our identification of a three-kringle domain containing protease in urochordates, the lower chordate closest to vertebrates, supports previous evolutionary theories regarding the ancestral forms of the PLG group in chordates [31, 34, 35, 37, 77–79]. As no PLG-related growth factor orthologues were identified in lower chordates, further functional characterizations of their PLG-like proteins are needed to clarify whether they possess dual activities as proteases and growth factors like the scenario proposed for *Caenorhabditis elegans*. Nonetheless, the canonical forms of both PLG and PLG-related growth factors appeared as distinct ancestors in all vertebrates. Interestingly, HGF in lampreys seems to be more similar to lamprey MST-1 than to the others HGF from jawed vertebrates (Fig. 4, Additional file 1: Figure S3 and Additional file 3) prompting the question whether HGF activity actually was present in the vertebrate ancestor or first appeared in jawed vertebrates*.* In two animal groups (coelacanths and birds) additional PLG-like genes appeared to lack some of the catalytic residues (Additional file 1: Figure S3) and may therefore represent a new potential class of PLG-related growth factors in those vertebrate groups.

### Emergence and diversification of PLG activators group

After examination of the different species belonging to the lower chordates, our results confirm that at this stage no orthologues resembling any member of the mammalian PLG activators family exists in this animal group (Table 2), thus corroborating observations from an earlier study [33]. Based on our analysis, we propose lamprey HABP2 as the most primitive member of the PLG activators group and as the sole representative of this gene group in lampreys. This is in contradiction to an earlier study that reported contigs encoding a trypsin domain resembling HGFAC in the genome of the sea lamprey in the Trace database archive [37]. Upon reexamination of the genome and transcriptome of several lamprey species (Additional file 2), we failed to confirm the existence of such an orthologue. A closer examination of the current accession of the contig in question (GL484904) from the UCSC genome browser reveals a 92% identity with the trypsin domain of lamprey HABP2. Accordingly, it clusters in the HABP2 group with lamprey HABP2 in the phylogenetic tree provided in Fig. 4. The presence of an extra FN2 domain in lamprey HABP2 (Additional file 1: Figure S2), yielding a domain composition half-way between HABP2 and HGFAC, suggests that this form may correspond to a common ancestor of jawed-vertebrate HABP2 and HGFAC. Such hybrid properties are well aligned to the model of evolution as hypothesized previously [34, 35]. Moreover, all jawed-vertebrates seem to have HGFAC (Fig. 3 and Fig. 4), which duplicated before the lungfish-tetrapods split giving rise to FXII in tetrapods, but being lost in the avian clade as previously reported [37, 80].

**Fig. 8:**
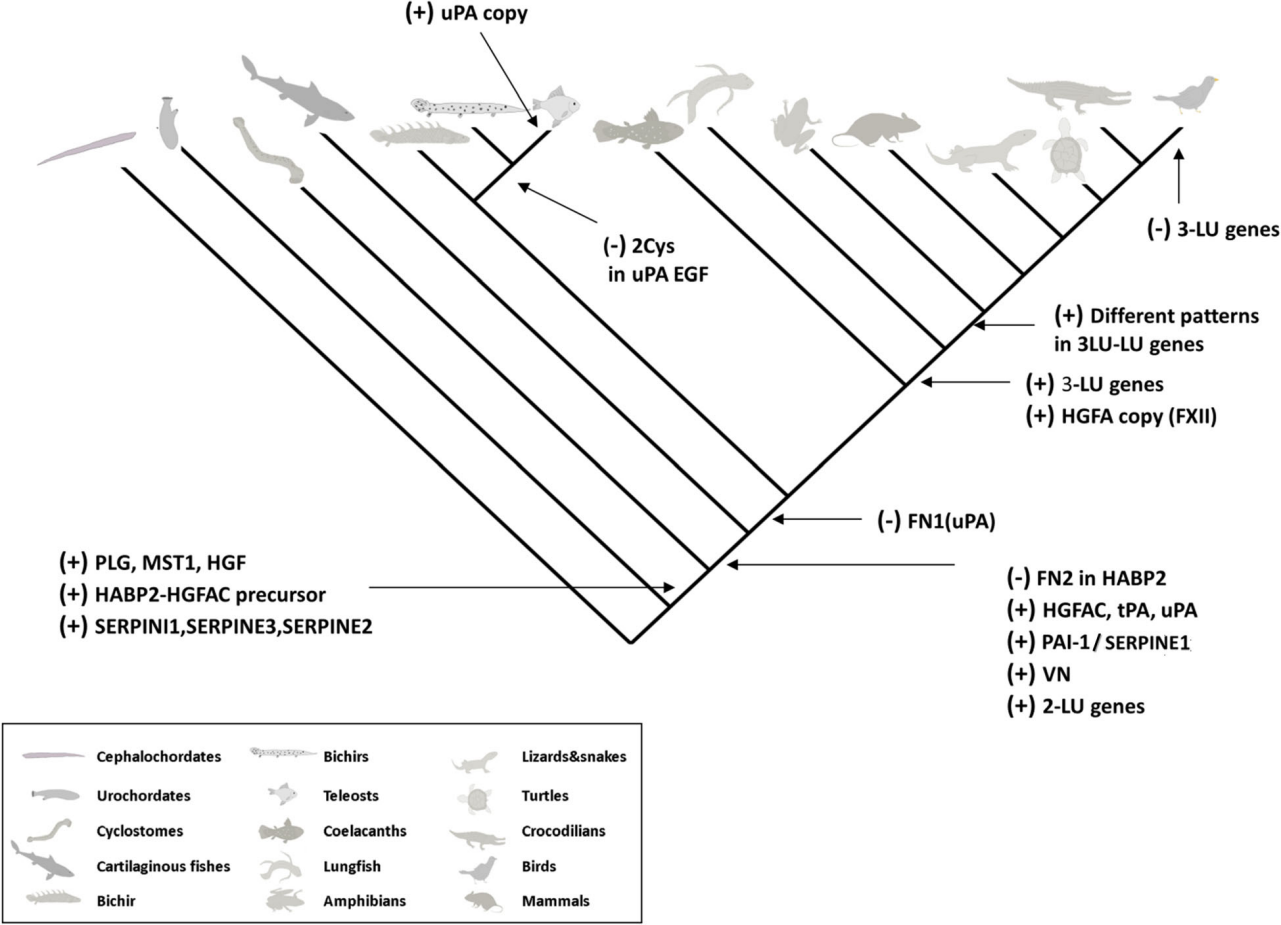
Overview of the appearance and diversification of the plasminogen activation system among chordates. PLG (plasminogen), HGF (hepatocyte growth factor), MST-1 (macrophage stimulating 1), HABP2 (hyaluronan binding protein 2), LPA (lipoprotein a), HGFAC (hepatocyte growth factor activator), tPA (tissue-type plasminogen activator), uPA (urokinase-type plasminogen activator), VN (vitronectin), FN2 (fibronectin type 2), LU (Ly6/uPAR domain), PAI-1/SERPINE1 (plasminogen activator inhibitor-1/serpin E1), SERPINE2 (serpin E2), SERPINI1 (serpin I1), SERPINE3 (serpin E3), FXII (coagulation factor XII/Hageman factor), Cys (cysteine residues)

Although an earlier study reported the presence of contigs resembling tPA in the genome of the sea lamprey (no accession was provided) [37], we failed to identify such an orthologue in the lamprey species we analyzed (Additional file 1: Figure S1). The closest possible candidate to tPA in lamprey would be a protease with two kringle domains, which by sequence similarity could correspond to a paralogue of lamprey HABP2 (HABP2-like-2) (Fig. 4 and Additional file 3). Phylogenetic analysis does cluster this candidate closer to lamprey PLG (Fig. 4). It would therefore appear that a primordial tPA gene is first detectable in cartilaginous fish and that it has been conserved in the entire jawed-vertebrate group (Fig. 4). In the case of uPA, origin and diversification also follow the model of evolution of modular serine proteases proposed previously [34]; it appears the first time in cartilaginous fish with an extra FN1 domain (and is thus more similar to tPA than the mammalian counterparts). During vertebrate cladogenesis that FN1 domain was lost (Fig. 8). Further diversification of uPA occurred within ray-finned fishes, where the loss of two cysteines in the EGF domain occurred before the teleost-gars split and hence before the duplication of the uPA gene (Fig. 5). This duplication occurred in teleosts and led to the subsequent loss of the EGF domain in one of the gene copies (Fig. 8) [24]. A canonical uPA structure is conserved from coelacanths to birds (Fig. 5 and 8).

### Serpin V3 group and VN

Serpins comprise a large group of more than 6000 proteins identified in eukaryotes, prokaryotes and archaea [38–42]. Comparative analysis of serpins from non-vertebrate model organisms might provide some clues to the origin and ancestry of vertebrate serpin genes. Recently, a novel serpin has been described in the invertebrate *Branchiostoma japonicum* [81]. While serpins from amphioxus are closely related to clade I of the group V3 serpins, our analyses did not provide evidence for serpin in the closest group to PAI-1 (V3 group, E clade). Among those five serpins that we focused on in this study, lampreys only present SERPINE2. According to our phylogenetic analysis and previous studies [38], SERPINI1 and SERPINE3 split from the main branch before the emergence of PAI-1 and SERPINE2 (Fig. 6) suggesting that the common ancestor of vertebrates contained SERPINI1 and SERPINE3. Interestingly, the SERPINE2 identified in the sea lamprey may represent a direct ancestor of SERPINE2 and PAI-1 in jawed vertebrates (Fig. 6). In accordance, the appearance of PAI-1 is coupled to the emergence of its predominant target proteases (tPA and uPA) as well as its cofactor VN (Fig. 3); and we recently reported that PAI-1 from a cartilaginous fish (*Squalus acanthias*) is not only able to inhibit uPA but also to bind to VN [26] confirming that already this primitive PAI-1 is functional. Interestingly, the shutter and VN-binding regions in crocodilians and bird PAI-1 are not as conserved as in other vertebrate groups (Additional file 1: Figure S5), which might suggest that in those animal groups, PAI-1 genes are might be under a different selective pressure.

The 37-loops of human uPA and tPA are enriched in positively charged residues, which are known to be important for the interaction with PAI-1 [82, 83]. In chicken, these residues are deleted in the uPA 37-loop and human PAI-1 is accordingly unable to inhibit uPA from this species [23]. Further verifying the functional importance of this deletion is the observation that by “humanizing” the 37-loop only, chicken uPA became sensitive to human PAI-1 [23]. It was therefore hypothesized that inhibition of uPA in chicken (and probably birds) followed a different molecular mechanism or that avian PAI-1 targets a different protease than uPA [23]. Notably, all avian uPAs studied so far are deficient of the basic residues in their 37-loops (Additional file 1: Figure S6). In conclusion, a pair-wise functional comparison of avian uPA and PAI-1 is required to clarify the functional role of PAI-1 in birds.

### Emergence of uPAR-like and 3-LU domain containing genes

In contrast to mammals, in which the only three-LU-domain protein present is uPAR, our data demonstrates the presence of several three-LU-domain containing genes in non-mammalian tetrapods. Remarkably, birds seem to have lost all three-LU-domain containing proteins. Although one gene comprising three consecutive LU domains is present in the ray-finned fish *Lates calcarifer,* it does not cluster together with any of the other three LU domain proteins (Fig. 7). Another peculiarity of this particular gene is that its second and third LU domains are almost identical (90% identity) and it is absent from all other teleost species investigated where only two-LU domain containing genes are found. We hypothesize that the extra LU domain arose as recent intra species domain duplication. Importantly, our RNA-seq data confirms the presence of uPAR-like genes with three LU domains in the lungfish, which clusters in the larger phylogenetic group of three-LU domain genes (Fig. 7). Accordingly, the two uPAR-like genes identified in lungfish represent the most primitive orthologue to mammalian uPAR—confining the evolutionary origin of the uPAR lineage to the common ancestor of lungfishes and tetrapods. Aligned with this proposition, the sequence alignment in Fig. 5 shows that lungfish uPA has a traditional EGF domain with a β-hairpin sharing some, but not all elements involved in uPAR binding in mammals [84]. Along with sequence similarity, domain composition and phylogenetic analysis, the homology of all the three-LU-domain containing genes with mammalian uPAR is confirmed by syntenic analysis in those species where a genome assembly is available (Table 3). In coelacanths a two-LU domain gene corresponds to the BRBH to human uPAR and this gene is located in synteny with the ETHE1 and XRCC1 genes as well. Based on this observation we hypothesize that a single domain duplication gave rise to a three-LU–domain containing gene after the coelacanth-lungfish split. Later in evolution, in the lungfish-tetrapod common ancestor, that gene duplicated and subsequently one copy lost a pair of cysteines in the first LU-domain, presumably engaged in the formation of a disulfide bond. Subsequent to the acquisition of the 8–10-10 pattern in uPAR-like proteins, another gene duplication occurred leading to a gene copy possessing an extra stretch in the C-terminal part at the end of the third domain (Fig. 7, Table 3 and Additional file 6). All three-LU-domain containing genes encode proteins which are predicted to be tethered to the cell membranes via a C-terminal glycolipid anchor as is the case for human uPAR [5], since they all possess the required C-terminal signal sequence for adding a glycosyl-phosphatidylinositol moiety. The only exception being the genes encoding three-LU-domain proteins with a 10–8-10 cysteine pattern (Additional file 6). Intriguingly, we were unable to identify any three-LU-domain containing genes in any of the analyzed avian genomes or transcriptomes. Pertaining to this observation, previous studies reported several specific gene losses (including uPAR, LYPD3 and XRCC1) in the avian lineage as a consequence of chromosomal rearrangements and the split of different chromosomes into microsomes [85]. Subsequent studies discovered that many of those gene losses in birds were enriched in conserved syntenic blocks [86, 87]. However, uPAR was not among those. Accordingly, we did identify several genes located in the uPAR gene cluster in different avian transcriptomes and genomes, but neither uPAR nor any other three-LU-domain containing genes. After careful scrutinizing the genome of the bird *Pseudopodoces humilis,* we identified several of the genes generally clustering with uPAR i.e. ETHE1, XRCC1, LYPD3 and PHLDB3 in the same scaffold (NW_005087786.1). Based on this evidence, we conclude that loss of uPAR, along with additional uPAR-like genes, occurred as a consequence of chromosomal rearrangements in the avian lineage. This is particular interesting since the growth-factor-like domain of chicken uPA contains all the known structural requirements for maintenance of a fully functional receptor-binding capability [84, 88].

## Conclusions

In this study we reexamined the molecular evolution of the plasminogen activation system genes through a combination of exhaustive mining of sequence databases and the generation of novel data by transcriptome sequencing. By sequence similarity, phylogenetic- and synteny analysis, we identified orthologues of the plasminogen activation system and related paralogues. Focusing on the serine protease members, we tracked their origin and diversification during the chordate clade and identified for the first time several ancestral forms which gave rise to the mammalian plasminogen activation system. These ancestral forms provide evidence for the step-by-step model of evolution by protein domain gains and losses established in earlier studies [34, 37, 78]. In addition, we have shown that after the appearance of all the PLG and PLG activators group members several gene duplications occurred, emphasizing the appearance of new members in this gene group previously unknown such as the additional copy of HABP2 and the appearance of a new class of PLG-related growth factor.

Our transcriptome data showed that lungfish presents the most primitive orthologue of mammalian FXII, pushing the evolutionary origin of that member of the contact phase of coagulation prior to the water-to-land transition during vertebrate evolution. We report that the origin of the uPAR lineage emerged before the appearance of tetrapods and that this was mirrored by the appearance of an uPA sequence compatible with receptor binding, as deducted from studies on mammals [84]. Notably, this phylogenetic comparison also reveals that plasminogen activation by uPA predates the evolution of a receptor-driven focalization of uPA-mediated plasminogen activation on cell surfaces. It is, nevertheless, still possible that a less efficient cell-surface associated plasminogen activation by uPA might occur in species lacking uPAR as the lysine-binding sites in plasminogen and the inhibitory properties of α_2_-antiplasmin would favor a surface associated state of active plasmin [52].

Therefore, although biochemical characterization of these variants is pending to further clarify the functional implications of our findings, we believe that the present study contributes substantially to shed light onto the evolution of the plasminogen activation system and provides a necessary evolutionary update for this proteolytic enzyme system.

## Methods

### Library preparation and RNA-seq for *R.marina*, *T.scripta* and *Protopterus sp*

All animals were obtained from commercial suppliers in Denmark. *T. scripta* were euthanized by an injection of pentobarbital (200 mg/kg), whereas *Protopterus sp* and *R. marina* were euthanized by submergence in water containing MS-222 (2 g/l). Euthanasia for scientific purposes, such as the harvest of tissue for in vitro studies, does not require permits from the animal care inspectorate. After dissecting, samples from brain, kidney, liver and gonads were collected from *T. scripta* and *Protopterus sp,* liver and kidney from *R. marina* and snap-frozen at − 70 degrees Celsius. Total RNA was extracted using mirVana miRNA Isolation Kit (Ambion) and depleted for rRNA using Ribo-Zero rRNA removal kit (Epicentre) according to manufacturer’s instructions. Sequencing libraries were prepared using ScriptSeq v2 RNA-Seq Library Preparation Kit (Epicentre) for strand-specific, multiplexed libraries and selected for an insert size of 300 bp. Paired-end RNA-seq was performed on Illumina HiSeq 2000 to a read length of 150 bp. The raw reads generated in this study are deposited in the ENA database under study accession PRJEB21481.

### Data processing and de novo transcriptome assembly

Possible remaining rRNA and mitochondrial reads were removed from the raw data by keeping the non-mapping reads from comparison the LSU_Ref and SSU_Ref Silva databases version 119 [89] and the mitochondrial genomes for these species using Bowtie2 and FastQScreen version v0.4.4 [90] with default parameters. Trimmomatic version 0.32 [91] was used to remove adapter sequences and trimming low quality bases using the parameters –phred33 ILLUMINACLIP: Scriptseqv2_adapters:2:30:10 LEADING: 3 TRAILING:3 SLIDINGWINDOW:4:15 MINLEN:50. Trimmed paired-end reads were combined for each species and assembled using Trinity software [62, 63] version 2.0.6 with default parameters except –max_memory 15G –CPU 20 –SS_lib_type FR –min_kmer_cov 2 –KMER_SIZE 25 –min_contig_length 200 –normalize_reads. Assembly metrics were obtained using the *Trinity_stats.pl* script from the Trinity software package. Reads were mapped back to the transcripts using the script *bowtie_PE_separate_then_join.pl* from Trinity software 2.1.1. Bowtie [92] was run with the parameters –p 20 –all –best –strata –m 300. The mapping percentage was calculated with the script *SAM_nameSorted_to_uniq_count_stats.pl* and the abundance of each transcript and gene was calculated using the *align_and_estimate_abundance.pl* script, both from Trinity software 2.0.6. From the latter, default settings were used except the options –est_method RSEM –aln_method bowtie –trinity_mode –prep_reference –SS_lib_type FR using RSEM version 1.2.19 [93] to calculate the expression. We removed transcripts with zero fragments per kilo base per million reads (FPKM), transcripts per million (TPM), and IsoPct (percentage of the reads that align to each isoform over the reads that aligned to all the gene isoforms). A second filter was performed to remove transcripts shorter than 300 bp. Predicted proteins were obtained using TransDecoder (https://transdecoder.github.io) with options –m 60 –S and annotated with Trinotate version 2.0 (https://trinotate.github.io) using SwissProt, Uniref90 and Pfam databases with the compatible Trinotate SQLite boilerplate. In addition, blast [94], hmmer [95], SignalP [96] and TMHMM [97] were used with recommended default settings. Completeness was assessed using BUSCO v1.1b1 [98] with the vertebrate dataset and the –trans option.

### Retrieval and analysis of data from public resources

In the case of the stingray *Leucoraja erinacea* the public RNA-seq reads were processed and assembled as described above. Regarding the three lamprey species, public RNA-seq reads (see Additional file 2 for details regarding species and accession numbers) were trimmed with Trimmomatic version 0.32 [91] applying default parameters. Trinity version 2.1.1 version was used for all the species except for *Petromyzon marinus* where the total reads were normalized prior to assembly. Previously assembled transcriptomes and complete protein datasets were downloaded from different sources (Additional file 2). The predicted sequences longer than 60 amino acids from de novo assembled and downloaded transcriptomes were extracted using TransDecoder with default parameters and protein sequence redundancy was removed at 90% identity with cdhit [99].

### Orthology identification

Blast reciprocal best hits (BRBH) between the human proteins and the chordate dataset were performed using blastp ncbi-blast-2.2.30+ [94] with an e-value of 1e-03 as cutoff. Further paralogues were investigated performing a similar approach but using as query the orthologues previously identifed for each species against their protein database and excluding self-hits from the results. Orthology assignment was refined by examining the protein domain composition of the hits using hmmscan [95] with an e-value of 1e-02.

Full-length sequence of lungfish uPA was generated after sequencing the open reading frame obtained from two overlapping contigs (primers pairs in Additional file 1: Table S2). The PCR products were cleaned using ExoSAP-IT cleaning kit (Affymetrix) following manufacturer’s instructions and sequenced on an ABI 3730xl sequencer.

### Generation of phylogenetic trees

Sequences were aligned with muscle [100] using default parameters. Alignments were trimmed with trimAl [101] with the option -automated1. In all cases, RaXML [102] was run with the predicted best model and the parameters raxmlHPC-PTHREADS -f a -× 12,345 -N 1000 -T 60 -p 12345. The best model was predicted using the option RaXML AUTO and running PROTTEST version 3.4.2 [103] with the parameters -S 0 -all-distributions -F -AIC -BIC -tc 0.5 -threads 25. If the models predicted for both methods disagreed, the model with the lowest Akaine information criterion (AIC) according to Prottest without invariant sites was selected. Accordingly, the model JTT + G was selected in all the trees. Finally, phylogenetic trees were visualized using iTOL [104]. The complete protein sequences used for the phylogenetic tree are provided in Additional file 7, Additional file 8 and Additional file 9.

## Additional files

Additional file 1: **Table S1.** Number of paired-end reads during the different filtering steps. **Table S2.** Primers used for sequencing uPA lungfish. **Figure S1A.** Diagram depicting the four principal theories about the evolution of plasminogen activation system. **Figure S1B.** Diagram depicting the four principal 488 theories about the evolution of plasminogen activation system. **Figure S2**: Protein domain composition of the serine protease member of the plasminogen activation system without canonical domain composition. **Figure S3.** Multiple sequence alignment of the catalytic triad from selected trypsin domains. **Figure S4.** Multiple alignment of the vitronectin N-terminal region in selected species of different vertebrate groups. **Figure S5A-C.** Multiple sequence alignment of selected PAI-1 orthologues. **Figure S6.** Multiple alignment of the 37-loop region of the trypsin domain of the uPA identified. (PDF 1998 kb)

Additional file 2: Accession numbers for Public RNA-seq reads, previously assembled transcriptomes and complete protein datasets were downloaded from different sources. (XLSX 65 kb)

Additional file 3: Unrooted maximum-likelihood phylogenetic tree with bootstrap values of the PLG and the PLG-activator group members identified in chordate species analysed. (TXT 47 kb)

Additional file 4: Unrooted maximum-likelihood phylogenetic tree with bootstrap values of serpin V3 members and PA1–2 orthologues identified in chordate species analysed. (TXT 23 kb)

Additional file 5: Unrooted maximum-likelihood phylogenetic tree with bootstrap values of uPAR-like genes and closely paralogues identified in chordate species analysed. (TXT 13 kb)

Additional file 6: Annotated fasta files of the three-LU domain proteins found. (DOCX 29 kb)

Additional file 7: Protein sequences of the PLG and the PLG-activator groups used to build the phylogenetic tree in Additional file 3. (TXT 415 kb)

Additional file 8: Protein sequences of the serpin V3 members and PAI-2 orthologues used to build the phylogenetic tree in Additional file 4. (TXT 136 kb)

Additional file 9: Protein sequences of the uPAR-like genes and closely paralogues used to build the phylogenetic tree in Additional file 5. (TXT 48 kb)

## Abbreviations

AIC: Akaine information criterion
Blast: Basic local alignment search tool
Bp: Base pairs
BRBH: Blast reciprocal best hits
C4.4A: Structural homologue of uPAR, also know as LYPD3
CD177: Cluster of differentiation 177
ECM: Extracellular matrix
EGF: Epidermal growth factor
ETHE1: Ethylmalonic encephalopathy 1
FN1: Fibronectin 1
FN2: Fibronectin 2
FSAP: Factor VII activating protease
FXII: Coagulation factor XII (Hageman factor)
HABP2: Hyaluronan binding protein 2
HGF: Hepatocyte growth factor
HGFAC: Hepatocyte growth factor activator
HX: Hemopexin
LDL: Low-density lipoprotein
LDLRA: Low-density lipoprotein (LDL) receptor class A
LPA: Lipoprotein(a)
LU: Ly6 antigen/uPAR-like domain
Ly6: Leukocyte antigen 6
LYPD3: Ly6/PLAUR Domain Containing 3
MST-1: Macrophage stimulating 1
mya: Million years ago
NGS: Next generation sequencing
PA: Plasminogen activation
PAI-1: Plasminogen activator inhibitor 1
PAI-2: Plasminogen activator inhibitor 2, placental type
PAS: Plasminogen activation system
PHLDB3: Pleckstrin homology like domain family B member 3
PINLYP: Phospholipase A2 inhibitor and Ly6/PLAUR domain containing
PLAT: Plasminogen activator, tissue-type
PLAU: Plasminogen activator, urokinase
PLAUR: Plasminogen activator, urokinase receptor
PLG: Plasminogen
PLG-2: Plasminogen 2
RCL: Reactive center loop
SMB: Somatomedin-B
SPN-1: Serpin 1
SRCR: Scavenger receptor cysteine-rich domain
SVH-1: HGF/MSP/plasminogen-like protein
TEX101: Testis expressed 101
tPA: Tissue-type plasminogen activator
uPA: Urokinase plasminogen activator
uPAR: Urokinase plasminogen activator receptor
V3: Gene structure-based phylogenetic classification group of vertebrate serpins
VN: Vitronectin
XRCC1: X-ray repair cross complementing 1
