# Supplementary material for: Origin and diversification of the plasminogen activation system among chordates
Source: BMC Evol Biol. 2019 Jan 17;19:27. doi: 10.1186/s12862-019-1353-z (PMC6337849; doi:10.1186/s12862-019-1353-z)
Supplement: Supplementary file 1 — Table S1. Number of paired-end reads during the different filtering steps. Table S2. Primers used for sequencing uPA lungfish. Figure S1A. Diagram depicting the four principal theories about the evolution of plasminogen activation system. Figure S1B. Diagram depicting the four principal 488 theories about the evolution of plasminogen activation system. Figure S2: Protein domain composition of the serine protease member of the plasminogen activation system without canonical domain composition. Figure S3. Multiple sequence alignment of the catalytic triad from selected trypsin domains. Figure S4. Multiple alignment of the vitronectin N-terminal region in selected species of different vertebrate groups. Figure S5A-C. Multiple sequence alignment of selected PAI-1 orthologues. Figure S6. Multiple alignment of the 37-loop region of the trypsin domain of the uPA identified. (PDF 1998 kb) [file 12862_2019_1353_MOESM1_ESM.pdf]

Additional file 1

SUPPLEMENTARY TABLES AND FIGURES:

Table S1. Number of paired-end reads during the different filtering steps.

Table S2. Primers used for sequencing uPA lungfish.

Figure S1A. Diagram depicting the four principal theories about the evolution of plasminogen activation system.

Figure S1B. Diagram depicting the four principal theories about the evolution of plasminogen activation system.

Figure S2. Protein domain composition of the serine protease member of the plasminogen activation system without canonical domain composition.

Figure S3. Multiple sequence alignment of the catalytic triad from selected trypsin domains.

Figure S4. Multiple alignment of the vitronectin N-terminal region in selected species of different vertebrate groups.

Figure S5A-C. Multiple sequence alignment of selected PAI-1 orthologues.

Figure S6. Multiple alignment of the 37-loop region of the trypsin domain of the uPA identified.

Table S1. Number of paired-end reads during the different filtering steps

Species	Tissue	Raw	Non ribosomal	Non ribosomal nor mitochondrial	Quality trimmed and filtered
Protopterus sp	Brain	164,294,354	163,808,838	139,976,826	130,446,916
	Kidney	222,432,954	222,045,632	211,381,084	185,622,950
	Liver	90,186,262	89,961,828	75,987,838	71,268,318
	Gonad	140,648,192	139,583,092	128,726,104	108,869,850
	Total	617,561,762	615,399,390	556,071,852	496,208,034
Rhinella marina	Kidney	82,867,074	82,828,232	81,104,332	64,894,856
	Liver	15,658,046	15,628,238	15,512,648	14,430,562
	Total	98,525,120	98,456,470	96,616,980	79,325,418
Trachemys scripta	Brain	88,584,156	88,558,894	46,116,490	39,878,946
	Kidney	115,962,760	115,828,190	67,979,928	47,636,654
	Liver	45,483,540	45,472,998	23,331,506	21,335,834
	Gonad	77,264,826	77,206,686	59,772,352	48,940,110
	Total	327,295,282	327,066,768	197,200,276	157,791,544

Table S2. Primers used for sequencing uPA lungfish

Forward	Reverse
AAGGACTACATTGGACAGAGAAA	TGCCTGTCCTATGTAGCCTT
GGAGGGTTCACTGGAACAGA	AGTTCCACCACACCGAAAAA
CCCATTGGTTTGTGAGAAGG	GCAGCTTCCTGTCCATATCC
CTGCCTCCATGGGATTTAAC	GCAGCTTCCTGTCCATATCC
CCCATTGGTTTGTGAGAAGG	TTCTGTTTGGCACACTCGTC
AAGCCATTTGGGAGAATTGA	GCAAATATGCAGCATGGAAA

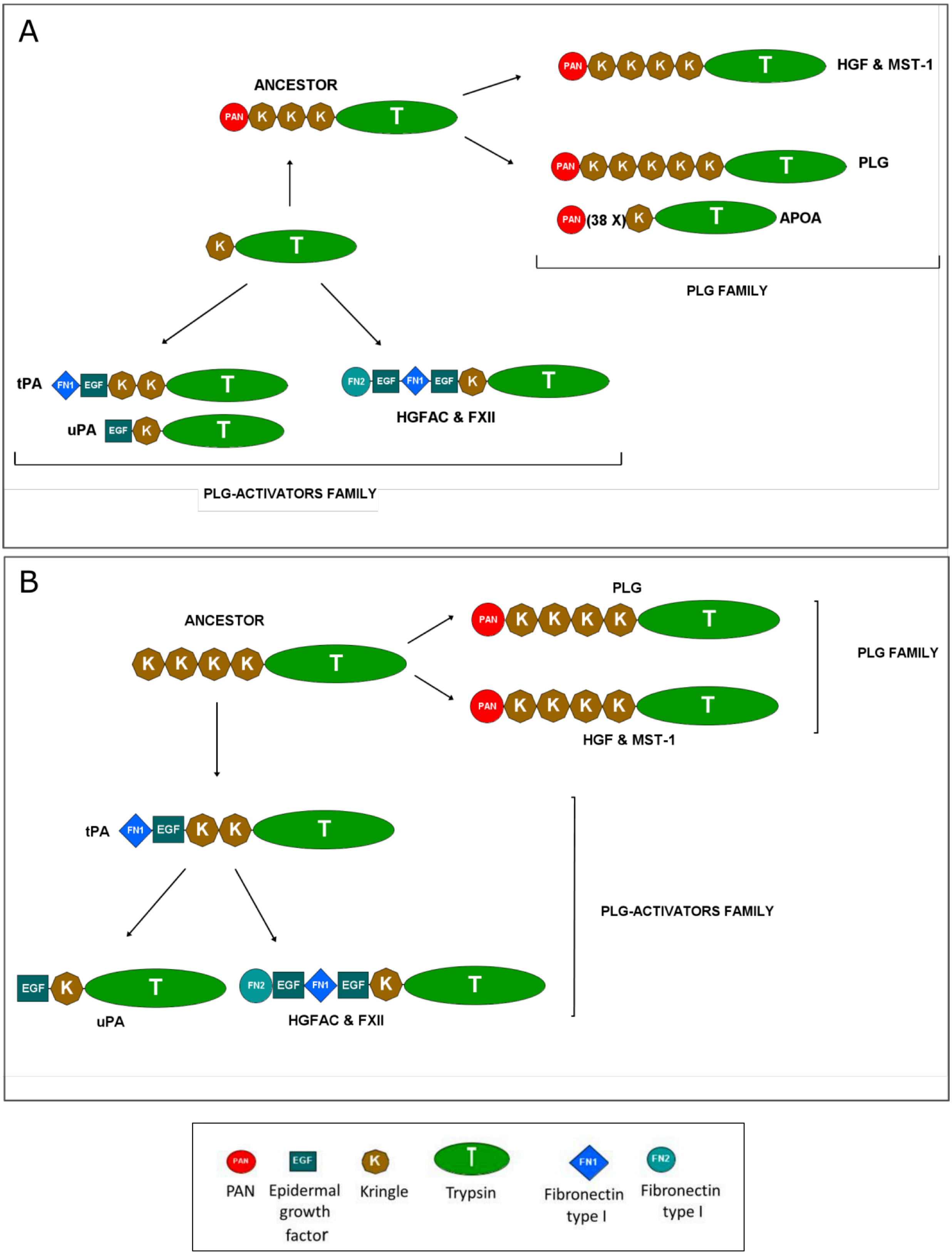

Figure S1A. Diagram depicting the four principal theories about the evolution of plasminogen activation system. Adapted from A: (Donate, 1994), (B), (Doolittle *et al*, 2009; Ponczek *et al*, 2008)

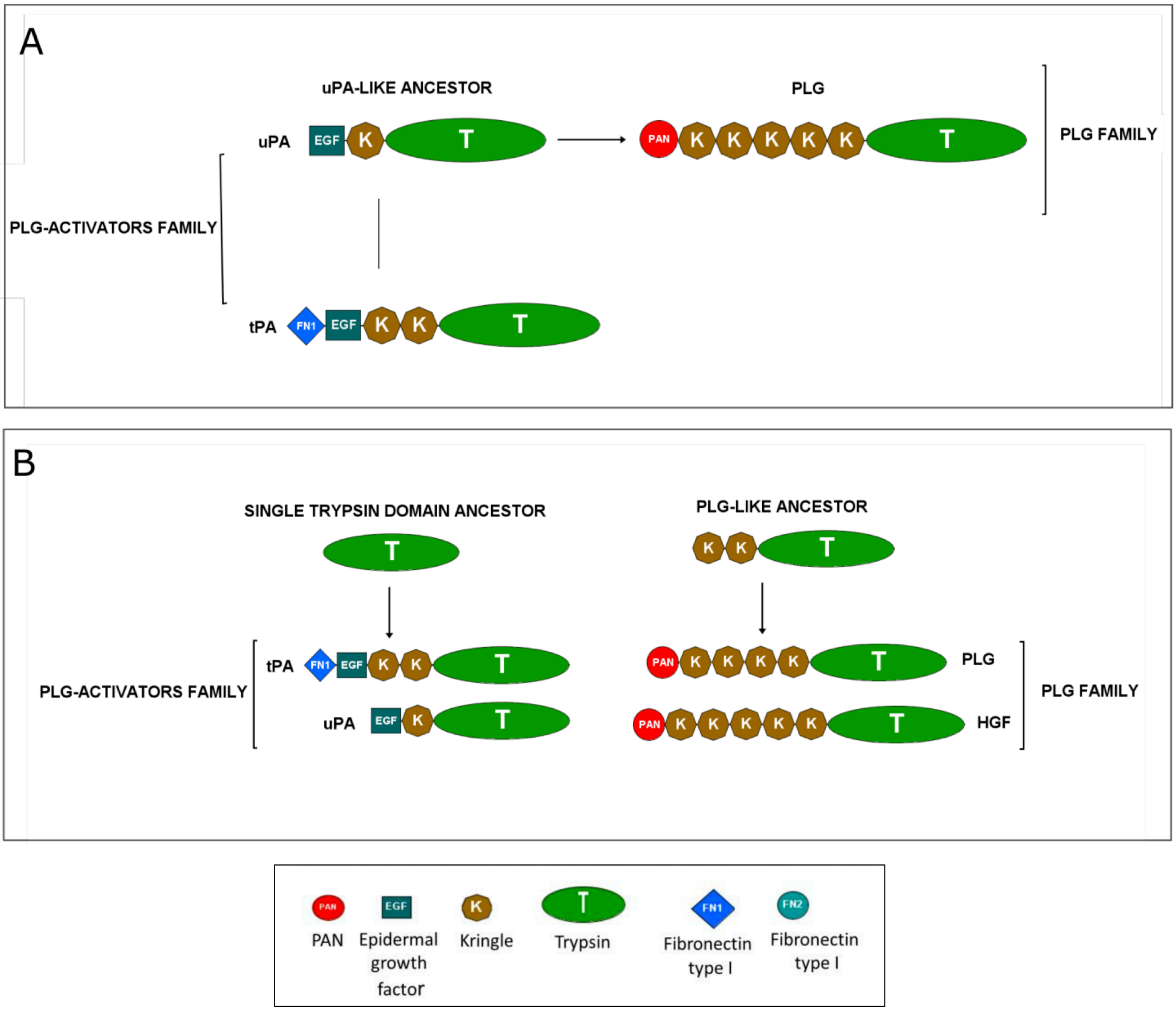

Figure S1B. Diagram depicting the four principal theories about the evolution of plasminogen activation system. Adapted from (A) (Ponczek *et al*, 2012) and (B) (Patthy *et al*, 1985)

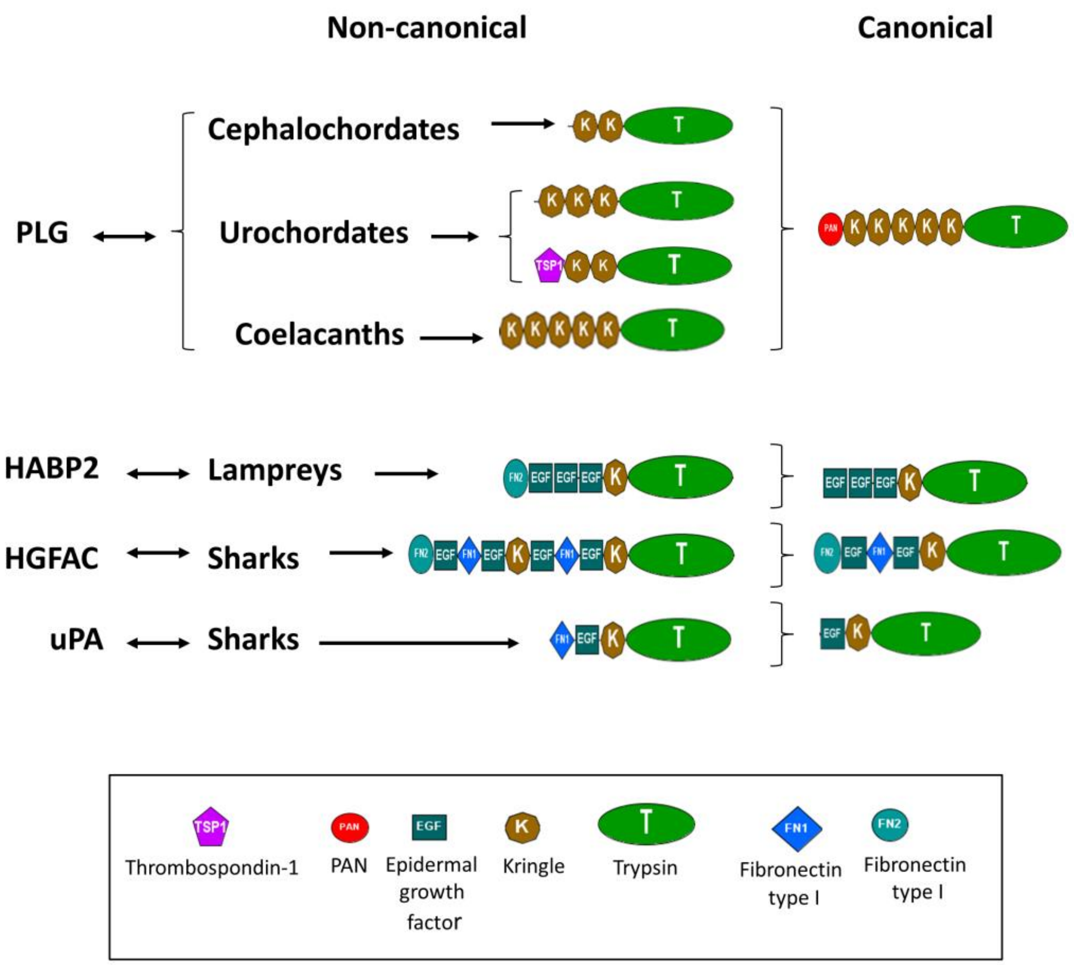

Figure S2. Protein domain composition of the serine protease member of the plasminogen activation system without canonical domain composition

```
plg_homo_sapiens                   -GGCVA-HPHSWPWQVSLRTRF------G--MHFCGGTLISPEWVLTAAHCLEKSPR-PS
plg-like_petromyzon_marinus        VGGCPA-KPHSWPWQVAIRKKLYSFSSQF--RPHCGATLIDPSWILTAAHCVNSS-D-VR
plg-like_latimeria_chalumnae       VGGCVS-RPYSWPWQISLRTRS------N--GCQCHYEKK------LTLLFLFRSSR-PA
hgf_homo_sapiens                   --------RTNIGWMVSLRY-R------N--KHICGGSLIKESWVLTARQCFPSR--DLK
hgf-like_petromyzon_marinus        ---------SHSPWTVSLRNRK------K--KHFCGGSLVDERWVLSARDCFVSCHGNVS
mst1_homo_sapiens                  ---------GNSPWTVSLRNRQ------G--QHFCGGSLVKEQWILTARQCFSSCHMPLT
mst1-like_petromyzon_marinus       --------PGDSPWTVSLRDGK------A--KHFCGAVLIDRLWLLSSRQCFPSCNLDAS
plg-like-2_latimeria_chalumnae     -------KPHSWPWQTSLQTSL------N--MHFCGGALIHPQWVLTAAQCYVRSAT---
plg-like-2_Nestor_notabilis        -----A-PPGSWPWHVSLRTST------A--EHHCAGSLIHPQWVLTAARCLQESTE-TS

plg_homo_sapiens                   SYKVILGAHQEVN---LEPH-VQEIEVSRLFLEP------TRKDIALLKLSS-PAVITDK
plg-like_petromyzon_marinus        LYRVLLGVHNELA---PEREAWQTVPVASIHTEP------RGVDIALVKIAT-PAQLTDR
plg-like_latimeria_chalumnae       AYKVVLGIHREHG---NEPS-RQERDVISILKGP------NRSDIALLKLNR-PVTLTDQ
hgf_homo_sapiens                   DYEAWLGIHDVHGRG-DEKC-KQVLNVSQLVYGPEGSDLV------LMKLAR-PAVLDDF
hgf-like_petromyzon_marinus        EFEAWLGMAKVKEGI--HER-MQAVPFSRMFCGPHGSKLV------MLKLSK-QVLLNGS
mst1_homo_sapiens                  GYEVWLGTLFQNPQH-GEPS-LQRVPVAKMVCGPSGSQLV------LLKLER-SVTLNQR
mst1-like_petromyzon_marinus       QYQAWIGIKKEDQVP-DNLN-SQSIAISKLVCGPANAKIV------MLKLER-AAFIDKD
plg-like-2_latimeria_chalumnae     YKVV-LGIHKKDG---NEPL-KQEFEVVKTFKGP------HTSDIALLKLNR-PALITDQ
plg-like-2_Nestor_notabilis        SYRVFLGIQSLNI---AEPS-LQIQSVQKVLKEP------SGADIALIKLSS-PVAITDR

plg_homo_sapiens                   ---------NGRVQSTELCAGHLAGGTDSCQGDSGGPLVCFEK--DKYILQGVTSWGLGC
plg-like_petromyzon_marinus        ---------NGRVGDTQFCAGYPEGGTDTCSGDSGGPLVSRDR--QRFVVHGVTSWGMGC
plg-like_latimeria_chalumnae       ---------NGRVSNNELCAGNIHGGADSCQGDSGGPLVCYDE--EKYIIQGVTSWGLGC
hgf_homo_sapiens                   ---------KVTLNESEICAGAEKIGSGPCEGDYGGPLVCEQH--KMRMVLGVIVPGRGC
hgf-like_petromyzon_marinus        ---------NRHIRQREICAGKGDRSGDACEKDYGGPLACVDN-NGRFVLQGVMPPAPGC
mst1_homo_sapiens                  ---------RGRVRESEMCTEGLLAPVGACEGDYGGPLACFTH--NCWVLEGIIIPNRVC
mst1-like_petromyzon_marinus       ---------KRQIDDTEICAGNTEGKIDSCERDYGGPLACREE--SCYVLQGIITPGRSC
plg-like-2_latimeria_chalumnae     ---------DGRVSDNQLCAGSIFSGTDNCKEDEGGPLVCQDG--DTFVLQGIASQILGC
plg-like-2_Nestor_notabilis        ---------NGSVRNHEFCGGFTFGSIENCEAEAGGPLVCQDK-DR-FVQYGVTSWGLDC
```

Figure S3. Multiple sequence alignment of the catalytic triad from selected trypsin domains. Catalytic triad residues are highlighted in yellow. The trypsin domains of lamprey (*Petromyzon marinus*) and *Homo sapiens* PLG group members are aligned against the new PLG-related growth factors identified in coelacanth (*Latimeria chalumnae*) and birds (*Nestor notabilis*. The trypsin domain of the PLG from coelacanth which has lost one catalytic residue is also shown. Only one coelacanth and bird species are shown for convenience in display. Full proteins sequences are contained in File S6.

```
mammalian_vtn_homo_sapiens                         QESCKGRCTEGFNVDKKCQCDELCSYYQSCCTDYTA---ECKPQVTRGDVFTMPED-EYT
sharks_vtn-like_squalus_acanthias                  --SCVGRCDLGFTPSEKCQCDSLCKFYKSCCEDYEH---ACK--KVRGDVFLMPED-EYD
bonyfishes_vna-like_danio_rerio                    -ESCIERCENGFDATKSCQCDSMCTYYKSCCTDYES---LCRI-KARGDTFPSHPEDDFD
bonyfishes_vnb-like_danio_rerio                    -ETCEGRCRSGFDPTKKCQCDRMCKYYGSCCEDFDT---TCRTKIARGDMFDLPEV-NVT
coelacanth_vtn-like_latimeria_menadoensis          --SCVGRCEEGFNSKEKCQCDTLCKYYQSCCADFET---TCNRKVSRGDVFTLPED-DYD
lungfish_vtn-like_protopterus_sp                   -DSCVGRCLRGFDKTKKCQCEPMCAYYESCCADYHT---ICKTKVGRGDVFNLPED-EYD
amphibians_vtn-like_rhinella_marina                -ESCIGRCLDGFDGGKKCQCDNLCIYYKSCCQDFIS---VCKPKETRGDVFSFPED-DFN
reptiles_vtn-like_anolis_carolinensis              -ESCEGRCDKGFDAARKCQCDDLCAYYQSCCEDYFT---TCRSKETRGDVFLQPED-DYL
turtles_vtn-like_trachemys_scripta                 -ESCVGRCEDGFHAQRKCQCDALCVYYQSCCSDYAM---ACKSKVTRGDMFAFPED-DYM
crocodilian_vtn-like_alligator_mississippiensis    -ESCVGHCDAAFNSSMKCQCDPLCVYYQSCCSDYNS---VCKTKVTRGDVFTFPED-DYS
birds_vtn-like_sturnus_vulgaris                    -DSCEGRCEEGFDAGHKCQCDTLCVYYQSCCSDYST---VCKAKVTRGDVFALPED-DYL
```

Figure S4. Multiple alignment of the vitronectin N-terminal region in selected species of different vertebrate groups. Only one species of each group was chosen as representative of the group. Cysteine residues are highlighted in yellow. Residues involved in binding human PAI-1 and uPAR (Zhou *et a*l. 2003) are highlighted in blue. Residues identical to the human binding interface in other species are in blue as well.

```
human_pai-1_homsa               -GVRVFQQVAQ—ASKDRNVVFSPYGVASVLAMLQLTTGGETQQQIQAAMGFKIDDKGMAP
tunicade_serpin_v3_oikdi        -MDLCLNLLK--SEPEKNLVFSSVNVEHAFAILATAAANKTREDILKMTSQADIAT----
shark_pai-1-like_squac          GIRIFREIAT--AAGDRNVVFSPYGAAALMGMAQLGAAGDTLAQLQNAMGYRLEDKGVPM
teleost_pai-1-like_danre        GLQVFAEAVQ--SAPDRNLALSPYGIASVLGMAQMGAYGATLKLLASKMGYSLQERGMPK
coelacanth_pai-1-like_latch     GIRVFQEVVS--SKGDQNVAFSPYGVTSVLAMLQLGAAGTTEEQLRTAMKYGLNEKGVAI
lungfish_pai-1-like_prosp       GIRVFNEIVK--SHRDRNVAFSPYGVASIMARLQLGAAGDTLAQIKEAMNYDVEERGVAS
amphibian_pai-1-like_rhima      GVRLFKEVLQ--ENKDKNLGFSPYGVSSALNILQYGTAGKTMAQVRQVLNYGYSERTVSA
lizard_pai-1-like_anoca         GARLFREATSAASSRHRNLAFSPPGVASVLAMLQAASGGGTRSQIEAGAGFRLRERGVPR
turtle_pai-1-like_trascr        GVKVFREVAK--VSPDRNVAFSPFGVASVLAMLQMAAAGESRSQIKAAMEYGVHERGIPQ
crocodilian_pai-1-like_allmi    GLRLFQAVVA---AAPGNAALSPTGAATLLRALQLVARGPGQAQLRTAAGYAPHEPGAGQ
bird_pai-1_stuvu                -GLRLFREALG—PRGDTNVVFAPYGATSVLVALQVATAGRGRQQLEEATGFSIDAPGVSA
```

Figure S5A. Multiple sequence alignment of selected PAI-1 orthologues. The PAI-1 homologue from the urochordate *Oikopleura dioica* is also shown. Shutter region residues in human and residues identical to those in other species are highlighted in red. Binding sites as seen in (Jendroszek et al. 2017)

```
human_pai-1_homsa                    ALRHLYKELMGPWNKDE-----ISTTDAIFVQRDLKLVQGFMPHFFRLFR-STVKQVDFS
tunicade_serpin_v3_oikdi             ---------TAHRVKSLESLSSVQLDSKMFTCF--QPKSEF---WKTHFTTASHGLVDFT
shark_pai-1-like_squac               ALKHLQKDLTAKSNQDI-----VHAANGLFVQRSMQLPRAFMKNYRKAFN-GWPKQVYFQ
teleost_pai-1-like_danre             LQRLLQRDLASE--------DGVEVASGVMVDRKIILEKVFRRSLSKAFQ-SVPHQIDFS
coelacanth_pai-1-like_latch          ALRQLKKALVSPWNRDI-----VSTVDAVFVQRDMELVNGFIKNFYRTFR-DMPKQVDFS
lungfish_pai-1-like_prosp            SLLNLKKSILAHWNKDV-----VKTADSMFVQRDMELVRGFYKQYYKTFR-DTIKQVNFT
amphibian_pai-1-like_rhima           ALRKLREEICGSLASGE-DTKSVHVADGLFIQRDLELTPGFLQRFHSTFR-RHVTQVNFT
lizard_pai-1-like_anoca              ALRQLQKALTEPRPEEEAEAVTVSTADALFVQRDLVLKAGFLPSFQRVFR-QAVKQVDFM
turtle_pai-1-like_trascr             ALRWLRKELTAPKNQDK-----VDVADALFVQRDLGLAPGFMKTFARAFR-QTVKQVNFT
crocodilian_pai-1-like_allmi         LEVAL-------------GGAGVALAAGIFVQHDLPLVPSTTV-----LA-PLLQRVDFR
bird_pai-1_stuvu                     ELRGLRRALRAPGQE-------LEVAQGLFVARGVALRPGFVTRLVRALGPRSLARLDFG

human_pai-1_homsa                    EVERARFIINDWVKTHTKGMISNLLGKGAVDQL-TRLVLVNALYFNGQWKTPFPDSSTHR
tunicade_serpin_v3_oikdi             DPKTA-DEINSWIEKSTKNMISKLVDASDLDSL-TRMILVSAIFFKGSWETPFRRTF--E
shark_pai-1-like_squac               DSEMATYIINKWVETQTRGMIPDFLRPGLLDPVQTRMVLVNAVYFKGLWKMPFPTEATHQ
teleost_pai-1-like_danre             QPEMARQVINSWTSDHTDGMISEFLPSGVLSEL-TRLVFLNALHFHGVWKTPFDPRNTRE
coelacanth_pai-1-like_latch          DQQRATYIINDWVKVHTEEMINDFLGPDTLDEM-TRLVLINAIYFKGLWKLPFPEEATHS
lungfish_pai-1-like_prosp            NLHQATYIINQWVKSHTEGMIPKFLSDDALGQL-TRMVLVNAIYFKGLWKLPFPEHKTRE
amphibian_pai-1-like_rhima           DSSQAKDILNQWVENQTDGMIQDLLGSNSIPPL-TRLVLLSAVHFDGKWVLPFPEKDTHE
lizard_pai-1-like_anoca              EPDRARSIINAWVEKHTEGMIQGFLREGLLDQL-TRLLLVDAIHFQGQWALPFPEASTRR
turtle_pai-1-like_trascr             EGERARFIINDWVKDSTHGMISDFLGPGTVDDL-TRLVLVNAVYFKGLWKLPFPEAATRQ
crocodilian_pai-1-like_allmi         HPASARRAINAWARTHTHGRIGELVGAGVLAGGAARLVLANTVHVSSAWARPFDPRATRP
bird_pai-1_stuvu                     FGEGARRGLNAWARSRTRGLVANLVAPGAVPAA-TRLLLASAESFRGSWGVPFPPRATRA
```

Figure S5B. Multiple sequence alignment of selected PAI-1 orthologues. The PAI-1 homologue from the urochordate *Oikopleura dioica* is also shown**.** Highlighted in green the human residues involved in the SMB-domain binding and as seen in (Jendroszek et al. 2017). Residues identical to that of human binding interface in other species are highlighted in green as well.

```
                                                                                P1   P1'
Human_pai-1_homsa                FTSLSDQEPLHVAQALQKVKIEVNESGTVASSSTAVIVSARM--APEEIIMDRPFLFVVR
Urochordate__serpin_v3_oikdi     YSNLTD-ESVFLSMARHKAKIEVNEEGTVAAAATVAKIMLKMCVIPIEFVCDRPFLYFIR
Shark_pai-1-like_squac           FTNISETEQLFVSKALQKVKIEVNESGTKASAATAAIFYERM--APLEVVIDRPFLFLVR
Teleost_pai-1-like_danre         FSRITTEEPLCVSKVLQRVKLEVNEEGTKGSSATAAVIYSRM--AVEEITLDRPFFFLIQ
Coelacanth_pai-1-like_latch      FAKISRAENLFVSQALQKVKIEVDESGTKASAATAAIVYARM--APLEVVMDRPFLFVVR
Lungfish_pai-1-like_prosp        FAKISRDELLFVSKGLQKVKIEVNESGTKASAATAAILYARM--APIEIVMDRPFLFVVR
Amphibian_pai-1-like_rhima       FSRLSTEKPLYVSEAFQKVKVEVTESGTKASSATAAILLARM--VPLEVIMDRPFLFIIR
Lizard_pai-1-like_anoca          FSSLSDEEPLFVAQALQKVKIEVNESGTKASAATAAIVYSRM--APLEMVLDRPFLFLVR
Turtle_pai-1-like_trascr         FTSLSAEESLYVAQALQKVKIEVNESGTKASSATAAIVYARM--APLEIIMDRPFLFVVR
Crocodilian_pai-1-like_allmi     LSGLTLDEPLFVSQALHKVKFEVMEGGTQAAAGTAAVFVARM--APPELALDRPFLFILR
Bird_pai-1_stuvu                 FSPVSGEEHLVLGQVLQKVRMEVTENGTEVASASAAVVYSRM--APLEIVLDHPFLFLIR
```

Figure S5C. Multiple sequence alignment of selected PAI-1 orthologues. The PAI-1 homologue from the urochordate *Oikopleura dioica* is also shown. RCL from P7 to P7’ is highlighted in blue. Protease cleavage site between P1 and P1’ is indicated by arrow. C-terminal residue of shutter region highlighted in red. Binding sites as seen in (Jendroszek et al. 2017).

sharks_uPA-like_callorhinchus_milii	VTIKSHPWQVAVYRKDRR-DENYFFHCGASLIHPCWVVTAAHCILPNT------LHTDYQ
sharks_uPA-like_squalus_acanthias	AIIESHPWQAALYITETR-STNPFFICGGSLIHPCWVLTAAHCIDTRV------SDAEYH
sharks_uPA-like_scyliorhinus_canicula	ATIELHPWQAALYVTDRR-SKDSYFFCGGSLIHPCWVLTAAHCIGTRV------SHEEYH
fish_uPAa-like_danio_rerio	STVESQPWMAAIFK--------GDGFICGGTLITPCWVLTAAHCFPTGKR----TQINRYS
fish_uPAa-like_lates_calcarifer	TPIESHPWVAALFHK-------RQGFLCGGSLISPCWVVTAAHCFVDGEV----TKTKYIS
fish_uPAa-like_cyprinus_carpio	SKVESQPWIAAIFK--------GVGFICGGTLIAPCWVLTAAHCFPGGKN----TQIKKYS
fish_uPAa-like_sinocyclocheilus_anshuiensis	SMVESQPWIAAIFK--------GDGLICGGTLIAPCWVLTAAHCFPGGKK----TQIKRYS
fish_uPAb-like_sinocyclocheilus_anshuiensis	SSVARHPWMAAV-----A--RGRAFTCGGSLISPCWVLTAAHCFPDGTK----TSIHKIS
fish_uPAb-like_fundulus_heteroclitus	AAVESHPWIAAIFWRTKS--KENVFRCGGTLISACWVVTAAHCFPEGSQ----ARHRRFS
fish_uPAb-like_lates_calcarifer	ATVESHPWVAAIFWRSKS--KEKVFRCGGSLISSCWVLTAAHCFPDGSN----TKERRFS
fish_uPAb-like_danio_rerio	SMLERHPWMAAIYSRK-S--RGRFFTCGGSLISPCWILTAAHCFPDGAQ----TLVHKLS
fish_uPAb-like_cyprinus_carpio	STVERHPWMAAVFSRS-A--RGRSFTCGGSLISPCWVLTAAHCFPDGTK----TSLHKLS
fish_uPA-like_polypterus_senegalus	ATIKSHPWMAAIYHHKRQ----EYFLCGGSLISPCWVLTAAHCFPPDAA----TKAHQYV
coelacanth_uPA-like_latimeria_chalumnae	AALESQPWIAAIFQYNHR-MKRDFFSCGGSLIDSCWVLTAAHCFPDLRNSRAHSSTNDFS
lungfish_uPA-like_protopterus_sp	TGTETQPWIAAIFKYSRR-DRATFFRCGGTLIHPCWVVTAAHCFPDSQ------KPNDFT
amphibian_uPA-like_ambystoma_mexicanum	APIESQPWISGIFKKSRS-AGQSYFQCGGSLIHPCWVLTAAHCFPDST------EPRDYS
amphibian_uPA-like_hynobius_chinensis	APIQSQPWISSIFKRSRK-QKQSFFQCGGSLIGSCWVLTAAHCFPEGK------EPSDFT
amphibian_uPA-like_rana_catesbeiana	SPIESQPWIAMLYQVSRRNRQQLFFQCGGSLIHPCWVVTAAHCFPDGET----PEPKDYA
amphibian_uPA-like_rhinella_marina	SPVESQPWIATLYQVNRRNRKEQFFQCGASLINPCWVLTAAHCFPDSEF----PEPKDYR
mammals_uPA-like_ornithorhynchus_anatinus	MSIDSQPWLAAIYRESRG-VE--KFICGGSLISACWVLSAAHCFTEHE-------KDHYS
mammals_uPA-like_monodelphis_domestica	TPIENQPWFAAIYRNHLG-GSS-SFTCGGTLISSCWVVSAAHCFPKIK------RGENYV
mammals_uPA-like_sarcophilus_harrisii	TPIENQPWFAAIYRRHPG-GSI-TFNCGGALISSCWVISAAHCFPKLK------RGETYL
mammals_uPA-like_mus_musculus	TEVENQPWFAAIYQKNKG-GSPPSFKCGGSLISPCWVASAAHCFIQLP------KKENYV
mammals_uPA-like_macaca_mulatta	TTIENQPWFAAIYRRHRG-GSV-TYVCGGSLISPCWVVSATHCFINYP------KKEDYI
mammals_uPA-like_pan_troglodytes	TTIENQPWFAAIYRRHRG-GSV-TYLCGGSLISPCWVISATHCFIDYP------KKEDYI
mammals_uPA_human	TTIENQPWFAAIYRRHRG-GSV-TYVCGGSLISPCWVISATHCFIDYP------KKEDYI
mammals_uPA-like_mustela_putorius	TTIENQPWFAAIYRKHHG-GSV-TYLCGGSLISPCWVLSATHCFINHP------KKEDYI
mammals_uPA-like_bos_taurus	TNAENQPWFAAIYRRHRG-GSI-TYLCGGSLISPCWVVSATHCFIDHP------KKENYI
mammals_uPA-like_ovis_aries	TTIESQPWFAAIYRRHRG-GSV-TYLCGGSLISPCWVVSATHCFTNHP------KKEDYI
reptile_uPA-like_gekko_japonicus	AEIESQPWIATIFQYMKR-NSAQRFLCGGTLIDPCWVATAAHCFKGRS-----PDPSQFT
reptile_uPA-like_anolis_carolinensis	AAIESQPWIATIFQSTRN-RGYNRFLCGGSLIDPCWVLTAAHCFESRT-----FDTSRLS
reptile_uPA-like_thamnophis_sirtalis	ASIESQPWIATIFRSRG-----NQFFCGGTLIDPCWVLTAAHCFPK-S-----SDTSKFI
reptile_uPA-like_protobothrops_mucrosquamatus	APIESQPWIATIFHSRG-----NQFFCGGSLIDPCWVLTAAHCFSGIG-----SDTSRLI
reptile_uPA-like_python_bivittatus	APIESQPWIATIFHTRG-----NQFFCGGSLIDPCWVLTAAHCFSGNG-----FDVSKLI
turtles_uPA-like_pelodiscus_sinensis	AAIESQPWIATIFHYSKR-TEKDLFICGGSLIDPCWVLTAAHCFSSGE-----KDPSGFK
turtles_uPA-like_chelonia_mydas	AAIESQPWIATIFHYSKR-MGQDKFVCGGSLIDPCWVLTAAHCFSQLG-----TDSSGYT
turtles_uPA-like_chrysemis_picta	ATIESQPWIATIFHYSRK-MGQNKFVCGGSLIDPCWVLTAAHCFSLLG-----TDSSGYT
turtles_uPA-like_trachemys_scripta	ATIESQPWIATIFHYSRK-MGQNKFVCGGSLIDPCWVLTAAHCFSPLG-----TDSSGYT
crocodiles_uPA-like_croco_porosus	AKIESQPWIGTIYHYSKK-AAENHFVCGGSLIHPCWLLTAAHCFQK-E-----INPSEYT
crocodiles_uPA-like_gavialis_gangeticus	AKIESQPWIGTIYHYSKK-AAENQFVCGGSLIHPCWLLTAAHCFQK-E-----INPSEYT
crocodiles_uPA-like_caiman_crocodilus	AKIESQPWIGTIYHYSKR-AAENQFVCGGSLIHPCWLLTAAHCFPK-K-----IYPSEYT
crocodiles_uPA-like_alligator_mississipiensis	AKIESQPWIGTIYHYSKR-AAEDQFVCGGSLIHPCWLLTAAHCFPK-E-----IHPSEYT
crocodiles_uPA-like_alligator_sinensis	AKIESQPWIGTIYHYSKR-AAEDQFVCGGSLIHPCWLLTAAHCFPK-E-----IYPSEYT
birds_uPA-like_Falcons_peregrine	AEVESQPWIAGIF-Q-TR-RGTDQFLCGGSLIDPCWVLTAAHCFHDPSN---PLDKSRYK
birds_uPA-like_Taeniopygia_guttata	AEVESQPWIAGIF-Q-TV-RGRDQFLCGGSLIDPCWVLTAAHCFHTP-----PINKSIYK
birds_uPA-like_Corvus_brachyrhynchos	AEVESQPWIAGIF-Q-NI-RGMDHFLCGGSLIDPCWVLTAAHCFHTPSR--RPINKSVYK
birds_uPA-like_Geospiza_fortis	AEVESQPWIAGIF-Q-TI-RGMEHFLCGGSLIDPCWVLTAAHCFHTPSR--RPINKSVYK
birds_uPA-like_Acanthisitta_chloris	AEVETQPWVAGIF-Q-TV-RGMDQFLCGGSLIDPCWVLTAAHCFHTPSR--RPLNKSAFK
birds_uPA-like_Apaloderma_vittatum	AEVESQPWVAGIF-Q-NV-RGVDHFLCGGSLIDPCWVLTAAHCFHSPSK--RTPPKSVYK

```
birds_uPA-like_Chaetura_pelagica            AEIESQPWIAAIF-Q-NI-RGIDHFLCGGSLIDPCWVLTAAHCFHTPSK--KPENKFLYK
birds_uPA-like_Cuculus_canorus              AEVESQPWVAGIF-Q-TI-RGTDHFLCGGSLIDPCWVLTAAHCFSTPSK--RPPNKFMYK
birds_uPA-like_Merops_nubicus               AGVESQPWIAGIF-Q-NI-RGVDQFLCGGSLIDPCWVLTAAHCFNNPSK--KPQDKSVYK
birds_uPA-like_Pelecanus_crispus            AEVESQPWIASIF-Q-TS-SGINHFLCGGSLIDPCWVLTAAHCFYTLSK--KPQDKSIYK
birds_uPA-like_Calypte_anna                 AEVESQPWIAGIF-Q-NI-RGIDHFLCGGSLIDPCWVLTAAHCFHNPSK--KPQ-KSAYK
birds_uPA-like_Nestor_notabilis             AEVESQPWIAGIF-Q-NI-RGVDHFLCGGSLIDPCWVLTAAHCFHSPSK--KPQNKSVYK
birds_uPA-like_Mesitornis_unicolor          AEVESQPWVAGIF-Q-NI-RGVDHFLCGGSLIDPCWVLTAAHCFNNPSK--NPQSKSVYK
birds_uPA-like_Eurypyga_helias              AEVESQPWIAGIF-Q-TI-RGTDHFLCGGSLIDPCWVLTAAHCFHNPTK--KPQDKSIYK
birds_uPA-like_Colius_striatus              AEVESQPWVAGIF-Q-NI-RGVDQFLCGGSLIDPCWVLTAAHCFHNPPK--KPQNKSIYK
birds_uPA-like_Phalacrocorax_carbo          AEVESQPWVAGIF-H-KI-KGVDHFLCGGSLIDPCWVLTAAHCFLAESK--QPRDKSIYK
birds_uPA-like_Picoides_pubescens           AEVESQPWVAGIF-Q-NI-RGVDHFLCGGSLIDPCWVLTAAHCFHNP-----PQNKPVYK
birds_uPA-like_Podiceps_cristatus           AEIESQPWIAGIF-Q-NV-MGNDYFLCGGSLIDPCWVLTAAHCFHNPSK--KPQDKSIYK
birds_uPA-like_Chlamydotis_macqueenii       AEIESQPWIAGIF-Q-NI-RGTDHFLCGGSLIDPCWVLTAAHCFHNPSK--KPQDKSIYK
birds_uPA-like_Haliaeetus_albicilla         AEVESQPWIAGIF-Q-NI-RGIDQFLCGGSLIDPCWVLTAAHCFHNPSK--KTQDKSIYK
birds_uPA-like_Haliaeetus_leucocephalus     AEVESQPWIAGIF-Q-NI-RGIDQFLCGGSLIDPCWVLTAAHCFHNPSK--KTQDKSIYK
birds_uPA-like_Egretta_garzetta             AEVESQPWIAGIF-Q-NI-RGVDNFLCGGSLIDPCWVLTAAHCFHASSR--KPLDKSIYK
birds_uPA-like_Buceros_rhinoceros           AEVESQPWIAGIF-Q-KI-RGVDHFLCGGSLIDPCWVLTAAHCFHTPSK--KPQDKSLYK
birds_uPA-like_Aptenodytes_forsteri         AEVESQPWVAGIF-Q-NI-RGIDHFLCGGSLIDPCWVLTAAHCFHSLTE--KPQEKSIYK
birds_uPA-like_Pygoscelis_adeliae           AEVESQPWVAGIF-Q-NI-RGIDHFLCGGSLIDPCWVLTAAHCFNTLTE--KPQDKSIYK
birds_uPA-like_Antrostomus_carolinensis     AEVESQPWIAGIF-Q-NV-RGIDHFLCGGSLIDPCWVLTAAHCFHYLSK--RPQDKSTYK
birds_uPA-like_Phoenicopterus_ruber         AEVESQPWIAGIF-Q-KV-RGTDYFLCGGSLIDPCWVLTAAHCFNSLSN--KPRNKSIYK
birds_uPA-like_Tauraco_erythrolophus        AEVESQPWIAGIF-Q-NI-RGTDHFLCGGSLIDPCWVLTAAHCFHTPSK--KPQNKSIYK
birds_uPA-like_Tyto_alba                    AEVESQPWVAGIF-Q-NV-RGINHFLCGGSLIDPCWVLTAAHCFHTPSK--KPQDKSIYR
birds_uPA-like_Pterocles_gutturalis         AEVESQPWIAGIF-Q-NI-RGVDHFLCGGSLIDPCWVLTAAHCFHTPSK--KPQNRSIYK
birds_uPA-like_Leptosomus_discolor          AEVESQPWVAGIF-Q-NI-RGIDNFLCGGSLIDPCWVLTAAHCFHNPSK--KPQDKSIYK
birds_uPA-like_Cariama_cristata             AEVESQPWVAGIF-Q-NI-RGIDHFLCGGSLIDPCWVLTAAHCFHNPSK--KPQDKSIYK
birds_uPA-like_Phaethon_lepturus            AEVESQPWVAGIF-Q-NI-AGIDHFLCGGSLIDPCWVLTAAHCFHTPSK--KPQNKPIYK
birds_uPA-like_Charadrius_vociferus         AEVESQPWIAGIF-Q-NI-RGVDHFLCGGSLIDPCWVLTAAHCFHTPSK--SPQNKSIYK
birds_uPA-like_Gavia_stellata               AEVESQPWIAGIF-Q-NI-RGIDHFLCGGSLIDPCWVLTAAHCFHTLSK--KPQDKSIYK
birds_uPA-like_Balearica_regulorum          AEVESQPWIAGIF-Q-NI-RGIDHFLCGGSLIDPCWVLTAAHCFNTPSK--KPQNKSTYK
birds_uPA-like_Nipponia_nippon              AEVESQPWIAGIF-Q-NI-RGVDHFLCGGSLIDPCWVLTAAHCFHAPSK--KPQDKSIYK
birds_uPA-like_Cathartes_aura               AEVESQPWVAGIF-Q-NI-RGIDHFLCGGSLIDPCWVLTAAHCFHTPSK--KPQDKSIYK
birds_uPA-like_Fulmarus_glacialis           AEVESQPWIAGIF-Q-NI-RGIDHFLCGGSLIDPCWVLTAAHCFHTPSK--KPQNKSTYK
birds_uPA-like_Struthio_camelus             AEVESQPWIAGIF-Q-NI-KGIDHFLCGGSLIDPCWVLTAAHCFHSQSK-----DKSAYK
birds_uPA-like_Tinamus_guttatus             AEVESQPWIAGIFER-NR-QGTYQFLCGGSLIDPCWVLTAAHCFRDGSK-----DKSDFK
birds_uPA-like_Anas_platyrhynchos           AEVESQPWIAGIF-Q-NI-LGIDQFLCGGSLIDPCWVLTAAHCFYDPSK--RRPRTSAYK
birds_uPA-like_Gallus_gallus                AEVETQPWIAGIF-Q-NI-MGTDQFLCGGSLIDPCWVLTAAHCFYNPTK--KQPNKSVYK
birds_uPA-like_Meleagris_gallopavo          AEVETQPWIAGIF-Q-NI-MGTDQFLCGGSLIDPCWVLTAAHCFYDPTK--KQPNKSVYK
birds_uPA-like_Sturnis_vulgaris             AEVESQPWIAGIFQT---IRGTDHFMCGGSLIDPCWVLTAAHCFHTPSRGP--INKSIYK
```

Figure S6. Multiple alignment of the 37-loop region of the trypsin domain of the uPA identified. Residues aligning to human 37-loop are enclosed in read boxes. Positively charge resides are highlighted in green.
