# Supplementary material for: Origin and diversification of the plasminogen activation system among chordates
Source: BMC Evol Biol. 2019 Jan 17;19:27. doi: 10.1186/s12862-019-1353-z (PMC6337849; doi:10.1186/s12862-019-1353-z)
Supplement: Supplementary file 6 — Annotated fasta files of the three-LU domain proteins found. (DOCX 29 kb) [file 12862_2019_1353_MOESM6_ESM.docx]

Fasta files of the three-LU domain proteins found. Highlighting representing signal peptides (red), cysteine residues (yellow), conserved Asn (cyan), signal sequence for GPI-anchor (green) and possible GPI-tethering sites (underlined).

>bonyfish_10-10-10-uPAR-like_lates_calcarifer

MHLLLIFGIVLLPKAYTLKCYECILGASGSCTGKEKECPSQDYQCGAMKVTSYTGESKLS

ADSKGCALPNECVEGSLNFGVARTVFTSKCCSSDLCNTQPAPDPSKSSPNGKKCFTCDGQ

KCTATLNCAGNEDSCISATVSVGGEKTTVKGCASKTVCANIQTAQMMEAIGAEMSCCQGD

YCNSANSTSAGFFSPSHWPHTPQTPFLILSTNPSKSSPNGKKCFTCDGQKCTATLNCAGN

EDSCITATVSVGGGKMTKKGCASKMMCANTQTAQMMEAIGAEMSCCQGDYCNSASSTSAG

LLLLVAPLISLVVFS

>lungfish_10-10-10-uPAR-like_protopterus_sps

MFLEIILFTLLSSAWSLQCYECQGLIDNCLISPVTCKPQEPYCILKAYRVIAGGITTDYL

MKSCSTGLECGQNTTLYTGNQAIYATSTCCEYDFCNAGAIQITVFSNYLQCYSCTGYKED

SCTSSSAINCIGVENQCVDVSTLVASGSSTTSSIIKGCGNGAFCNDLQAYNTGDSFSVVN

VTCCNTAKCNNRQFSASLPTNINGIRCYSCYETGKNECSANNKSQVNCTGGLYRCMEVLD

NNNGKQVALMRGCATESFCRGVIPSLTVPQSQQVLCCTGNLCNNWIFNGYQQTGKGHCLQ

TLNILYLLILLLPSVTVL*

>lungfish_10-10-10_protopterus_sps

MKIIWVVLYMGTLLYVAASLECYECSGNSTTCPEVRRTCGPAEDVCITVSQRSIYTGRAK

VELLTKSCSSSVSCIGNMSFTAASLSYSAITNCCKTNLCNDGKFFLPDSTAPNGLTCTSC

YGGTLGDCDGNDKPTINCTGPDNQCINIDIKWESIGLFTAKGCGSLPFCNDIIGIQQITG

LVGCCNTNNCNNATTTIIPTIKNNQKCYSCLKIDNSACNPIQAACYGSMTKCGDISTKVT

ANGFPLDIFATGCISPPLCHLQSALATVGLDSTTQFECCNGNLCNVKNFIFPTSPPTTTR

NAVSTTIKTSNALTIVIFLSVVFIT*

>amphibian_10-10-11b_ambystoma_mexicanum

MKWGWTLVAFCGLLTPGGCLECLECYGLPYNCTERVRECYAHQKACISKAYTYNENGVTT

DLLIKGCSQGLACNESAYVNLGTRAMYTSNTCCTSNQCNRGIYYARATVSQVTCKSCLNN

SIACSSPTLPTIHCAGVQDLCMSMTTITITQGTRMETVVKGCGTGPLCGRELDYYTGNTT

IYSKVECCQSNDCNTGVPRVTENNTRNGLRCYACNDTGKGECNSTRNVLAQLNCTGQMTR

CLDIVGFPSGKTLMRGCCSEKVCLGLSPSLSIPSFQNLYCCTGNLCNNGSITSYFTLPSH

GRILRTSGASLACLLLLTTALRWALQK

>amphibian_10-10-11a_ambystoma_mexicanum

MKAALGTLYILIFFPTVFSLKCFQCRGSSASCRMVRQRCLAYETTCISLAYQSTTPLLTT

NSVMKGCASTTLCNVTSAIDTGTNSFYMTSTCCETDYCNSNRFSAVGVVSKQLQCSSCVD

AETCKTPTQLACRNTNNQCVDVVTEIYNNGTMTNSSIKGCGSGEACGALLSYNTGRSQLY

TQFQCCSSNRCNNRTISVSKPRFPNGIMCWGCNETGNNECAPEKQQMVKCSGSLVRCMEA

FGPDRKTLKKGCCTEDLCSDTYPAVSGIPKEVEIQCCAGNLCNQWGKVYSCPEDYSAGWR

LRGSPGLLLAVGYAGLLRIIL*

>amphibian_8-8-10-uPAR-like_ambystoma_mexicanum

METAFSCLLLCALLTQACCLECFKCDGSANCTMGTERCWEGQNWCQAILTQWTTGNDTQR

SVVKGCAWMEGTNQTTSYSSKQTRSMQSEYYCSADLCNREDPAGALGADVPNGFQCHTCS

SLVTCNETENSIQNCTGLEKQCVFSSQKLMFPDRMSMMKSVSKGCWHNPGSAGAVGYITA

HSSLLISFCNSSLCNNATMGFQHQNRSVNGVTCFTCEGNRTTSCSHQNEALIECYGPLTQ

CMEAMSVDEQNNVTSIIKGCATPAWCQHPLLTGFQKEDFHSFQCCEGRLCNTGMANTTEP

DALSTTASTSSMPRSSNVRGNGAENGGASVSGSLQFLVALLLISTSWNLMMATGFN*

>amphibian_10-10-11b_tylototriton_wenxianensis

MRGAWTLVVFCGLLQPGLCLECLECYGLPYNCTETVRECYAHQPTCISTAYSYEDASGMK

ELLVKGCSQGLSCNESAYVNMGTKAVYISNTCCSRNQCNWGTYYAKATVSQADCAVCSGD

SASCSSPTLRVMHCTGVQDLCMSITTLTIYRGVARETVVKGCGTGPLCGRNLEYNTGNTT

LYSKVECCRPSNCKPQVPTVSVNNTQNGLQCFACNETGKNECNATRITSVNCTGQMNRCL

DIVDFGQGKTLMRGCCSKDVCQGLPASLSVPSYQSLYCCQGNFCNTGNIASYFSGGRSLE

VSGASLAGILLLGMAWWWAL*

>amphibian_10-10-11a_tylototriton_wenxianensis

MKAVLWTLLLFLPVAFSLKCLKCQGSSSYCRMRKQTCAAYETTCISLAYLSKGSTTVDTV

MKGCSTTALCNTTSALSIEASSIFMTATCCDSNYCNFNSFSAAKVVSNELQCQSCTTSTG

CETPNNVTCRETNNKCMDMATLEGNNGTLTGTYVRGCGSGEACGKYMAYDTGITQQYSLV

KCCNSINNCNDKRIVVTKPPYHNGITCWGCNESGKNECALENQSVVRCNGSLARCFQMFG

GPNRQTLKKGCCTEELCSNIYPAVQGIPANTEIQCCAGNLCNKWPNTYPCPEDYSACRRP

WRNPGLLVATLCISVLSRLL*

>amphibian_8-10-10-long_tylototriton_wenxianensis

MELRWICHVLLLLSASWTTVNSLECYGCEGDAECSQSPISCHGPDAYCQTSVLTASVFFL

TALNIKKTCAYGERPDKVTQLNAKQKVSLSLQENYCNTNFCNNETDFELVPAEPNNLHCY

GALCQGQHCASSTAMDNLMCRGNQTKCVELAISGKMGLTSDVSMKGCAEVPECEEEMGFR

STHASFHLQCCDSDFCNGGTVPNDDDLMPNGVVCYSCNEGDEKIGCPLERATMVNCTGSM

SSCLEASGVIRESDVVSSRVIRGCATPKTCSGSFLSLIQQFQEPQVFCCSGNLCNSRTFV

GVQNASHGVSDKPSSGGLFQHSSHVPSNADIFSDANTANASVIATTSMQRNSNLTLSDNE

NIMNSQSEMSILSYYGDSTAHLETGNNSDLMQSEYQQYSGYDSQQQTGIQTIDDLILSHV

GNPLDYLDLNVSVSNMSSDLYQNDVDKGSYSTDGAGSVFQSLKPSGSNNSIFSNSSTVFL

SGPNNIAISNDNGSASVSGGNKNNSLSNSSAIYHSGANDTGLSSNDYNFAVNSGMALEGS

NDLNYKNLSTVSPIASSTASPVAPTFSTIVSGSTTSDHISEILSGGTNGSAAFNSSLDYN

GSSDHVGLNGSTSPSVIPPGAPNSTISPSSTTLYSGLDHTDISTGNRSVYNTSSVGHDVT

THILISSAGDNSSAHPAGIYSGIHSVSLSSSISTVYHNVSEHTTPSPGNHSTGSFSAMPE

ESHNVSVSNSYSVIYHNGSGPSDHDTSTHSSTSHSGDIAGGNLLVSNNSTTVTPMSTVFS

TMLSGNQTGLSKLGISSGVRNGSGVFNSSMVYHSNSNHTGHDNDTTHPLMSSGGHSDLIH

NTSATHYYSGSDINIVNGSSASGSGSNSTTTNYGEFAQTTIPPSDHFIDITVSEPILSYP

HSSHGEVASTSPPTNTESSTSHFGMSVGETNGSTYNTSSGDYNDSVSVMPPGGHNVTISN

ISSTIYHSDTEHTGVSTVDHNNSSALEISPGGHNESLLNISSAVYHSGSEHTYGSLSGMP

PGSHNGSISNASSDTHHSDSEHIGFSTSDRNNGSLSGMTSVGHIDSDHNTSSIMYHSGLE

HVGLSTVDHINGSHTGMPTNLNNTTHFNISSDSEHIDLPTGDYKNDSFSGMPFEGNNYSL

SNTSSNSGLSSVDDNSNSLSGMLSEGRNDSLSNMSSNIGLSPDDYNSVSFLGMPPGGHND

SLSNISSTVYHTGSEYNGLSTVHHNNDSLSGIISDGHNDSLSNISSNIYHSGSEHAGNYT

GDHSNSSPTEVPSVGHNDTISNTSSTIYHSDFEHTGISTPDHSNGSLPRMPSEGHNNSVS

NSSALYDNSSDHFNFSISGLDSALLTGAFSGNRNSSSNSSVPVSHVNPNHTNMANGHSSV

SHSDITSTSNNGSLLGYHTGTNHTDISPWGNESSSVNMSVSSLGANSHSFLNGNSSTMNT

SDSHHASSSGISSTDDHGNVSMHDSSVETVSSSFFDSAVNSDLIYRGNLNDTSGLTMALT

TTMKGETGNNVDTTNGHPGRIVNTYVFSAVFIVGLMY*

>amphibian_10-10-11_xenopus_laevis

MSKWLSLLTLCFFLPVVFSLKCYQCTGLPYDCQETEQLCQINQTSCMSQAFTDVDNGTIK

EWTYKGCSQGLVCNESAYLDRGSRKTYISSSCCSTNFCNVGTYYALVPVAALYCKSCEGN

SDSCSSQNLTSVQCSGFHDRCMTVTTVFNNGTLSSESAIKGCGTGNLCNRSLEYNSGNGR

IYTSVSCCGKTNCNTGRNTVTFNETLNGLMCYACNETGKGECRTPTTVSCTGNMSSCLDI

IGFPRGNTLIRGCCSQDVCLGISASMSVQVSQKQYCCSGNLCNNGDINSYFSTGSIMAHS

NVSIMGIALLVLTVVTRTML

>amphibian_10-10-10_xenopus_laevis

MGKKWRILLLLINFWQVDSLRCLQCIGSASSCRMVAKKCPAYENTCISLAYRSTAVSTTS

NTVMKGCSTRALCNQTSVIDTGNRSIYMSASCCETDYCNINRYSTKAVFSNRMQCYTCKN

NNIYCGYPNLDKVFCDGVDNWCVDVLTKEYTNGKVTASSYTKGCGSGEACNSLLAFDTGS

FQRYTQYSCCNSQTLCNNAYQSIPMLSNINGITCWACLDTGNNECDVKNQVQISCKGTLI

RCMEAYDQNRQVVMKGCSTVTYCSTTYPSLNVSNIAEIQCCAGSNCNNFTQMSTTEISGA

WSPSTDIRLLILLFALLGTVFCIVDPN

>amphibian_10-10-11_uPAR-like_nanorana_parkeri

MISFVEKQEKGLADDTDDLSTGRRLLIFCLPPWTKRTAAVGPCLPLKCNQCTGRPFDCVE

STQLCTLNQTSCMSQSFTYVENGTSTEWTYKGCSQGLRCNESVYVDMGYRKTYISSQCCI

SDMCNSGTYYARVPVAVLYCQTCQGNSASCASTNLTTIQCAGVQDRCITLSTVYNNVSSS

DVVIKGCGTGNLCGRRLHYNTGGVKVYSEMSCCGYNKCNQGVNTVTVNTLVNTTLNGLQC

YACNETGKGECTTPTTVSCTGSMTSCLDVLGYPRGNTLMRGCCSTDVCYGLSASMSLQAS

QKLYCCNGNLCNNGKITSYFSSGTTMARGRVYIIAGALLVLVELLRTLL

>amphibian_8-10-10-long-uPAR-like_xenopus_laevis

MGIIILLFGFLVLVDPGNALSCYNCTSEKGCKGKEVTCPGKNDTCSTSVLTISAFPLSYR

SVFKSCLNTPRPSRASFFTPSRTVRFSLQETTCSSDLCNNQTKFDFVEKINDLNCFSCIS

PGKSCDSDMMRKLQCRKEQKQCVDLRIKGTLGDISDTSLKGCGYFPACNGDLLFSNQKTS

LSVKCCGDNFCNSQTDYPTPKKLPNGKQCYSCQTDDGKKCSAENSQKTKCYGDLTSCVEM

AGLSMQGGTPKVMILKGCANPSMCKSTALPLLQKLNNATVQCCNDNLCNKKFTEDNFLTD

GNPPIKPTPTSIPPSTPTATLPDMNFSNSSMNNSIYSTSSPIQNPGISSNNSGLVGNSTL

NNTSLTTQNNTSVQSSPLPTGNDTTVNGTNNALPGGNNSGNGSATVGNASAPITSFPVTN

TSAGNTSASNQTLPGLSNAGVSNTSVNGSSNASNASNASNAGNASVPTVSSPTVLNAGGG

NGSTCNNSACSVIVPVNSSATGNMSSGANNVGNGSNNTSNLGNSSHSNTSSSLSNSGAVN

NSTANIPGSNGTASGLNQSGPGITGGGGVLNGSNASSNGSSSTGNVNGSMGNSTNTNVNN

GSVTSITGAGNGSSVNDSGIIGSVSNPVQSPPGSSNGSSPGSSSTGNATSTNVNNGAGNG

SSVNGSSNGSSPGLSSTGNSTSTNVNNGAGNGSSVNGSSTGSSPGSSSTGNSTSTNVNNG

AGNGSSVNGSSNGSSPGSSSTGNATSTNVNNGTGNGSSVNGSSNGSSPGSSSTGNATNTN

VNNSAGNGSSVNGSSNGSSPGSSSTGNATSTNVNNGAGNGSSVNGSSNGSSPDSSSTGNS

TSTNVNNGAGNGSSVNGSSNGTIPGLSSTGNSTSTNVNNGSVTSITGAGNGSSVNDSGII

GSVSNPVQSPPGSSNGSSPGSSSTGNSTSTNVNNGAGNGSSVNGSSNGSSPGSSSTGNIT

NTNVDGPRNNTASTTSEGLTTGFVTGGSSNVNTTDVPKIDNQTTNGKGDTFTTSIFILMT

AMLLNVLNSL

>amphibian_10-10-11-uPAR-like_xenopus_tropicalis

MHKWLSLLILCFFLPVVFSLKCYQCTGLPYDCQETEQLCHINQTSCMSQAFTVVDNGTIK

EWTYKGCSQGLVCNESVYLDRGSMKTYISSACCSTDLCNVETYYALVQVAGLYCQSCEGS

SDSCSLQNLTSVQCSGVQDRCMTVTTVFANETVSSESAIKGCGTGNLCNRSLEYNSGNGR

IYTSVSCCGKTNCNTGRNSVTFSETLNGLMCYACNETGKGECKTPTTVSCTGNMSSCMDV

VGFPRGNTLMRGCCSKDVCMGLSASMSIQVSQKQYCCSRNLCNNGNINSYFSKGSIIARS

RGVMGVALFILTVVLRTL

>lizard&snake_8-10-10-uPAR-like_anolis_carolinensis

MDFFLISIVLLLTVPHISGLQCYSCDRDTDCTEMEICEEHQEQCSTTIMTVLSRPKISTY

ILKGCDVSGKPNNSISHLSGNQVVFLTEEYCDTELCNKRSPNVVDVLIARGRQRRTRECY

SCTTADKTCSNSSLELMTCARLEEDCVDIISFTTELPAGEQRIKGCGQLSHCQASEPLGF

HNQNSFHLIKCCNSSRCNSDMQDYKDAPLPLNGVTCFSCEGNSTHGCSPDAVSKIQCQGP

MTQCLEASGIHGISGENSVVKGCASPSWCESPYTAVHKNLGAIHSRCCSGNLCNNWIING

TLKPSPRSQAGHTFQAQQTLLSTALLLSVTFLLCSGSS

>lizard&snake_8-10-10-uPAR-like_gekko_japonicus

MNFFSWIRFFFLFSLVAYVLGLQCYSCDGDSNCLEARVCPEHQDRCRTTVMTTLTRSGIS

TYYHKDCDVSGKANNSVSYLSHNQVVFLAEEHCESELCNEHAPNVLDVLLARGRPPNLKQ

CYSCSSADESCFNSTLAQMRCSRPGEQCVDITSFTVPEEFSQDELHIKGCGQLSHCQETL

GFHNQDSFYLIKCCNSSLCNKETQDYKASPLPLNGATCYSCEGNFSHGCAPGDITQEQCR

GPMSQCLEASGIDGVSGQSSIVKGCASSSWCNSPYTSIYKNLGAPYTRCCTGKLCNNQIV

DETTLKPSARSQACPNLTAGPVLLSAGLLLWMALLLSSETS

>lizard&snake_8-10-10-uPAR-like_protobothrops_mucrosquamatus

MKGFWTGVLFSLVVTEVVALQCYSCSGNSDCRETETCKEHQKMCKTTVMTIINRHKISPY

FLKGCDVSGKPNNSISHLLGHRLVFLAEEHCETDLCNSGVPKEVPRMRDMIHVRGHRENI

LSCYSCTAADNTCNNSSLTQMNCFWPQEKCVDITSLTDPEEFPKDQERIKGCGQLSHCQD

ALGFQNHKSFHMINCCNSSWCNKHVQDYKQTPLPLNGVTCHSCEGNTTHGCAPDNITTVQ

CRGPMTQCLEASGIHGISGNNSLIKGCASPSWCDSPYTSIYKNLGSVETHCCANNLCNSW

IIDGQMKASPRSQASHITLVQHTLISTSFLLFIVFLLS

>lizard&snake_8-10-10-uPAR-like_python_bivittatus

MECFWTSVLFSLVVADVVGLQCYSCHDHSDCQETEVCEEHQEKCRSTVMTIVVRPKISTY

FLKGCDVSGKPNNSISHLSGDRVVFLAEEYCETELCNGGVPKEAPRVGDMIRARGRNDNI

LSCYSCTAADKTCNNSSLTQMRCFWPKERCVDITSLTDPEESPRDQERIKGCGQLSHCQE

TLGFHNHKSFHMIKCCNSSWCNNDMQDYKKDPLPLNGVTCHSCEGNTTHGCATDNIAKMQ

CRGPMTSCLEASGIHGISGKNSVLKGCASPSWCNSPYTSVYKNLGSVETHCCVGDLCNSL

IIDGQLRPSPRSRASRFILAPQTVISTGLLLFVVFLLSSESS

>lizard&snake_8-10-10-long_anolis_carolinensis

MARFLAVLLLPALFNQAFCLQCNTCHGEYNCIGENVTCENPYASCTTSVRKAYVSFLEFQ

SVRKGCAQQLYPPESISIKSHLMSLSYQARFCAEDGCNNETYFVSHQPPANHMRCHTCAS

QGAWCPEIARTQISCSGHQDQCVDLTILGKLGQYSNLKIKGCSSLKRCEDTLSFYSGMRT

IHASCCNSPLCNTFSTDFHVLSEAPNGLECYSCVDDDGSGPGCTTQAMSKVQCTGIHNMC

LEGVGNSRKAGKDLGLVTFKGCASPAMCQSSLLGLVQELDNTDVLCCQGSLCNNRIVNGI

VTEARIPADSMDIAEAPECIKPSQAPSGVTAVPDCAYTEEKENDGVVSVHPSAGIHIEEE

TNVNERENNGKLVTENNTMSHFPHHDDHSITHKDTITENISEGSSQGNAASPSGAPGSDL

ITGDGSSAATILDKSNPGASTTSSGNHGNVVVLIPVVVSRRNNTTSSSTETTTDNRILAR

SNTNDEIDYEECEEEVEGMTTGEHFMAAKESNHDGSTSTPRSTIVPVEAHSDAGIFVEGM

NRDESAYLPSSNQGGHGAGIFTREGSTHASATDPAGSSNVVHEHNVHAHGRDDGRFTVDE

TTANHAPGPGDSTVDNHGATSGENFFGGSPPLQPGRVSDTIIPIPFVVSKDNDSFITEGN

NVANTNSPVATSGPDRDILVSGAGAGTARPKNKIPCKRPGSQRRQGAKLVSSDATNKRVS

EEVFTRDGVTSLFSDTKNPSHSGGGKVNPNSGSLGLLNNLNLFLLSLLMVALLH

>lizard&snake_8-10-10_python_bivittatus

MAKILIMLLLLTLLSQVFCLQCNTCHGEYNCVGENVTCDDPSASCTTSVRKAYVSFLEFQ

SVRKGCARQFYPSESISLTSHLMSLSYQAHYCMEDGCNNETYFVSHPAPTNHMRCHTCAS

QGAWCPEASRTQLSCVGNQDQCVDLDITGKLGHYSNLKLKGCTNLPRCEDTLSFYSGGQT

IHASCCDTPLCNTFTPDLHAQSQAANGLECYSCVDEEGSGAGCTNQTISKVQCTGINSMC

LEGIGNSRKAGKDVSFVTFKGCASPAMCQSSLLALVQELDNADIICCQGNLCNDRIVDGV

ITIPRVPADSPEYFEEVECATPSPTPSGLIPDPDCFYSDEAEDGTMVVVQSPTGEVPSPG

NHTDGETVYLADEKEASENFVNESDTRPSSQHDHVFAGGNPYEENIFGNSSESNASSPTG

VSYSEHGSSGSSSAITTLDTSNSSSSPPSSGNYGGVAVLIPFIIPKRNTTNTATTSSSIE

TGASKEVLTASTNDNDSASEECEAEEEGTTTGEHFMVANENDHEGSPSTPPASSFYESGT

PENTSSSAEDTPNAGVFVRGENNNELESYSSSTENTYNVSVLTTEEHGSPHSPVTDTSGS

TNMEGGHGATGDKNFVVDNSSLVSAPTSNSLPSDGIASNILPISNSSFITEGKTGTSRPK

NKIPCKWPGSQKLPGVNLVSSSGTGSPVTEEDAIRNAGTSSYSNAKSPERNAAGKVNLDS

RALGLSSHFGLFSLTLLLAALIPLVC

>lizard&snake_10-8-10_python_bivittatus

MKAAPASSILLLALLTTASGLTCYKCLGTRENCTNQEQICTENETVCMEETRATYGPMSE

TLKRGCTTPQHCQLYFAVRRGFVSRSIYCCSQDLCNAVPYNVTYTAKRNGLECYSCIGSL

GECSGTDVPTAQCRGRENHCVEISRQWLPETGLVEPIIKGCGDYPKEERLLAYSIGSETS

YVAIRICEGSKCNNNSFPEFLTREPNGLKCYSCLEEGNGECNIENLQLADCMGTMDQCMT

VLDYTHNTRIRAGCANQEFCQDTTFYGALMPKLPISYMICCQSPFCNGQPGQGISSTRVL

LSLLMVPLFIALNQQTVTSLQITSL

>turtle_8-10-10-uPAR-like_trachemys_scripta

MKSVWGCVLLGALLSQVAALRCYSCDGDQSCQETEDCGEQQGWCRTTILTMIAHSEVSKR

IQKGCDVEGKPNNSISFFSHGQIVMLAEEQCATDLCNQGVPDALRSFPPTSSLDCLSCSS

SDHSCSGPSMTRIRCLDPREQCVDITAISMAEEFPQDERRIKGCGQISQCQEPLGFHNRD

NFYLLQCCNSTLCNNDAHDYEKSPLPLNGVTCYACEGNSSHGCAPENVTPVQCQGPMTQC

MEAVGSHEWTGPGTVLKGCATPAWCDAPYTSVYKRLAGVQAHCCRGNFCNSRMQAGALPR

SRAGHGLAAQPALLCGTALLA

>crocodilian_8-10-10-uPAR-like_alligator_mississipiensis

MPIMRGLWIRIGLFVLVALGTAAALRCYSCEDEENESCPQLDCAPGQDRCRATAMASLRP

LTRTAQWVRTSRGCDVAGKPNNSVSFASTTGSVLFLSEHFCASDLCNREDPRGPDFPHPR

GTPLRCVSCAASEGSCSGRDLAPLPCADPRDQCVDIATLGGPHDAPDERRLKGCGRLWGC

GAPLGFHSAGSLFLLRCCNRSLCNDDPHDVEVSQLPLNNVTCYSCEGNRTHGCAPHALAE

LHCRGPMTRCLEAEGTHEVEGPGWRVRGCAXPAWCDSPYTAIYKGLQGAGAQCCQGDRCN

QGAAPPWPPATALLAPLGLLLIQLP

>crocodilian_10-8-10-alligator_mississipiensis

MTPTLYMVMLLGLLVTGGWPLPPPYSQPQLLSLLHPVVHPQNHLHLPTASGLRCPKCLPW

QGNCKGKVEMCVPGQNACVVQQLRLTSVMENALRVGCRKCEVLPSDTEAGFSVTSTCCFT

DLCKAIDMSLPSLALGPPCQSCVGSATTCGPNSPTVTCGDPRGQCVQISRRLLPGEKGDT

MYKACGWQGPSEELLAIAAGPDLAYVHVQRCRGAGCNNGSFAEVPRGKPNGKLCYTCRDT

GAGECNRRQMPTMSCTGAMDQCVKVRSTDWNKPAILLRGCGTPNLCGPLQPRGRLLLPFE

RKVHCCSGNHCNYEAPLPASRARPPAAPGTAAAGGAWS

>crocodilian_8-10-10-long_alligator_mississipiensis

MGGTLRALILGLALLGALMVPAWSLTCKQCHGEFGCDVESEVMCAPGEDACRTAVRTAAV

SFMQFQAVTKGCARGGARPEEALELRGHLFALATRTQHCAHNHCNDDVLLAAPPAAPNAL

HCHSCSVHGPGCPPTARTLLGCTGAQDRCFDLALRGVLGSFSDMRLKGCALLPGCDHGAL

GFDTGSGALRAQCCDTPFCNHQDSDLHLEAEAPNGLHCLSCLDQDGSGCPTDTDAGATVQ

CTGAHTVCLEGVGRTRRGTPSGTLVTFKGCATPAMCQSSLLALVQELDDTQVLCCNGSLC

NRHLPRGVLVAASTAPQPTGVPNCMPCTSTPTLPPIPKPDCIQEDNDHTGPRPGTATTMG

GHGATLPPSTERSWDRMLVPGGFYSDDSTARTADEQGHPAWEAAGNYITAAPQGTGSSRD

QVLHAGGFYSNNNTVTSTGGQNQPGREVSDNHDTASSQSADSSWDRVLNDSGVYSNGNTM

TATSRQGWEASGNHVTASSRGSDSSRDQVLDSGGFYSSGNTETDTGRHGQPGWEVSGNHI

TASSQGVDSSWDQVLNAGGVYSNGNTVTATFGRGPPGWEVSGNHITASPQGTDNSRDRVL

DTGGFYSSGNTVTATGGHDQPSWEASGKHVTVSSQDTDGSQDRVLNAGGFYSDGNTMMAT

GSRNQPGQEVLGSYITASSQGMDNSRDQVLDAGRVYSDGKTVTATGRHDQPGWEVSGNHI

TASPQGSDDSRDRVLDAGGFYSSGNTVTVTVGHGQPGREASGSVAPAMVAEQECVDDAEG

TTSVSRTAGVSAPVGSKVMASPSPHPVQPQDPLAAILPVPYLFPRQMEGGASEAEGRAMV

TPGTRRPCRRRPPGKGHMVAWEAGGARDSASSSTHMFPINIRNITAATSFPGQSRPGLPE

WGRNETWAKEPGLGKPNPPLPSGGPGPTPGLCLLGLTALLGALLC

>mammal_8-10-10-uPAR_homo_sapiens

MGHPPLLPLLLLLHTCVPASWGLRCMQCKTNGDCRVEECALGQDLCRTTIVRLWEEGEEL

ELVEKSCTHSEKTNRTLSYRTGLKITSLTEVVCGLDLCNQGNSGRAVTYSRSRYLECISC

GSSDMSCERGRHQSLQCRSPEEQCLDVVTHWIQEGEEGRPKDDRHLRGCGYLPGCPGSNG

FHNNDTFHFLKCCNTTKCNEGPILELENLPQNGRQCYSCKGNSTHGCSSEETFLIDCRGP

MNQCLVATGTHEPKNQSYMVRGCATASMCQHAHLGDAFSMNHIDVSCCTKSGCNHPDLDV

QYRSGAAPQPGPAHLSLTITLLMTARLWGGTLLWT

>mammal_8-10-10-uPAR-like_bos_taurus

MGQPLLLLLLVYTYIPGSWGLRCLQCENTTSCSVEECTPGQDLCRTTVLSVWEGGNEMNV

VRKGCTHPDKTNRSMSYRAADQIITLSETVCRSDLCNKPNPGRDATVSRNRYLECASCSS

TDLSCERGWDQTMQCLKSRDQCVDVITHRSLKENPGDERHIRGCGILPGCPGPTGFHNNH

TFHFLRCCNTTKCNAGSVLELQNLPPNGLQCYSCEGNGAHRCSSEETFLIDCRGPMNQCL

EATGTKGLRNPSYTIRGCAAPSWCQSLHVAEAFDLTHVNVSCCTGSGCNHPARDDQPGKG

GAPKTSPAHLSFFVSLLLTARLWGATLLCT

>mammal_8-10-10-uPAR-like_macaca_mulatta

MGHPLLLPLLLLLHTCVPASWGLRCMQCKSNGDCRVEECALGQDLCRTTIVRMWEEGEEL

ELVEKSCTHSEKTNRTMSYRTGLKITSLTEVVCGLDLCNQGNSGRAVTVSRSRYLECISC

GSSDMSCERGRHQSLQCRSPEEQCLDVVTHWIQEGEEGRPKDDRHLRGCGYLPSCPGSSG

FHNNDTFHFLKCCNTTKCNEGPILELENLPQNGHQCYSCKGNSTHGCSSEETFLIDCRGP

MNQCLVATGTYEPKNQSYMVRGCVTASMCQRAHLGDAFSMHHINVSCCTESGCNHPDLDI

QYRKGAAPQPGPAHLSLTITLLMTARLWGGTLLWT

>mammal_8-10-10-uPAR-like_mus_musculus

MGLPRRLLLLLLLATTCVPASQGLQCMQCESNQSCLVEECALGQDLCRTTVLREWQDDRE

LEVVTRGCAHSEKTNRTMSYRMGSMIISLTETVCATNLCNRPRPGARGRAFPQGRYLECA

SCTSLDQSCERGREQSLQCRYPTEHCIEVVTLQSTERSLKDEDYTRGCGSLPGCPGTAGF

HSNQTFHFLKCCNYTHCNGGPVLDLQSFPPNGFQCYSCEGNNTLGCSSEEASLINCRGPM

NQCLVATGLDVLGNRSYTVRGCATASWCQGSHVADSFPTHLNVSVSCCHGSGCNSPTGGA

PRPGPAQLSLIASLLLTLGLWGVLLWT

>mammal_8-10-10-uPAR-like_mustela_putorius

MGRPRLLPLLLLLVQTCVPASWSLQCLLCGRTGKCQVEECARGQDLCRTTTLRIWEGGEE

LEVVERGCAHPEKSNRTMSYRTGTQIITLTEALCATDLCNQPSPGRTSTFPRTRNRYLEC

VSCASSDLSCERGWDQSLQCRSPTEQCVEVVTHRSLEGSPRDEHHTRGCGNLPGCPGPTG

FHNHHTFHFLQCCNTTKCNGGSVVELQNLPLNGLQCYSCEGNSTHGCSADESSLTACQGP

MNQCLEATGTNGLGNPIYTVRGCATPSWCQSLHVAEAFSLTHLNVSCCTGTGCNSAPRLG

PAHLSLTTTLLITARLWGGTLLWT

>mammal_8-10-10-uPAR-like_ovis_aries

MDQKPLLLLLLVHTYIPASWGLRCVQCKNATSCSVEECASGQNLCRTTVLSVWEGSNKMN

VMRKGCTHPDKTNRSMSYRAGNQIITLSEAVCGSDLCNKPNPGPDATFSRNRYLECASCA

STDLTCERGWDQSMQCLKSRDQCVDVITHRSLKENPEDERHMKGCGILPGCPGPTGFHNN

HTFHFLRCCNTTKCNAGPVLEIQNLPPNGLQCYSCEGNSAHRCSSEETFLIDCRGPMNQC

LEATGAKGLRNPSYTIRGCAAPSWCQSLHVAEAFDLTHVNVSCCTGNGCNHPARDAQPRK

GGAPQTSPAHLSFFVSLLLTARLWGATLLCT

>mammal_8-10-10-uPAR-like_pan_troglodytes

MGHPPLLPLLLLLHTCVPASWGLRCMQCKTNGDCRVEECALGQDLCRTTIVRMWEEGEEL

ELVEKSCTHSEKTNRTLSYRTGLKITSLTEVVCGLDLCNQGNSGRAVTYSRSRYLECISC

GSSNMSCERGRHQSLQCRNPEEQCLDVVTHWIQEGEEGRPKDDRHLRGCGYLPGCPGSNG

FHNNDTFHFLKCCNTTKCNEGPILELENLPQNGRQCYSCKGNSTHGCSSEETFLIDCRGP

MNQCLVATGTHEPKNQSYMVRGCATASMCQHAHLGDAFSMNHIDVSCCTKSGCNHPDLDV

QYRSGAAPQPGPAHLSLTITLLMTARLWGGTLLWT
